# Contact-dependent growth inhibition (CDI) systems deploy a large family of polymorphic ionophoric toxins for inter-bacterial competition

**DOI:** 10.1371/journal.pgen.1011494

**Published:** 2024-11-26

**Authors:** Tiffany M. Halvorsen, Kaitlin A. Schroeder, Allison M. Jones, Disa Hammarlöf, David A. Low, Sanna Koskiniemi, Christopher S. Hayes

**Affiliations:** 1 Biomolecular Science and Engineering, University of California, Santa Barbara, Santa Barbara, California, United States of America; 2 Department of Molecular, Cellular and Developmental Biology, University of California, Santa Barbara, Santa Barbara, California, United States of America; 3 Department of Cell and Molecular Biology, Uppsala University, Uppsala, Sweden; Tufts University School of Medicine, UNITED STATES OF AMERICA

## Abstract

Contact-dependent growth inhibition (CDI) is a widespread form of inter-bacterial competition mediated by CdiA effector proteins. CdiA is presented on the inhibitor cell surface and delivers its toxic C-terminal region (CdiA-CT) into neighboring bacteria upon contact. Inhibitor cells also produce CdiI immunity proteins, which neutralize CdiA-CT toxins to prevent auto-inhibition. Here, we describe a diverse group of CDI ionophore toxins that dissipate the transmembrane potential in target bacteria. These CdiA-CT toxins are composed of two distinct domains based on AlphaFold2 modeling. The C-terminal ionophore domains are all predicted to form five-helix bundles capable of spanning the cell membrane. The N-terminal "entry" domains are variable in structure and appear to hijack different integral membrane proteins to promote toxin assembly into the lipid bilayer. The CDI ionophores deployed by *E*. *coli* isolates partition into six major groups based on their entry domain structures. Comparative sequence analyses led to the identification of receptor proteins for ionophore toxins from groups 1 & 3 (AcrB), group 2 (SecY) and groups 4 (YciB). Using forward genetic approaches, we identify novel receptors for the group 5 and 6 ionophores. Group 5 exploits homologous putrescine import proteins encoded by *puuP* and *plaP*, and group 6 toxins recognize di/tripeptide transporters encoded by paralogous *dtpA* and *dtpB* genes. Finally, we find that the ionophore domains exhibit significant intra-group sequence variation, particularly at positions that are predicted to interact with CdiI. Accordingly, the corresponding immunity proteins are also highly polymorphic, typically sharing only ~30% sequence identity with members of the same group. Competition experiments confirm that the immunity proteins are specific for their cognate ionophores and provide no protection against other toxins from the same group. The specificity of this protein interaction network provides a mechanism for self/nonself discrimination between *E*. *coli* isolates.

## Author summary

Bacteria compete for growth niches and other limited resources in the environment. Many species of Gram-negative bacteria use CdiA effector proteins to inhibit the growth of neighboring competitors. CdiA proteins carry a variety of toxin domains that are delivered directly into target bacteria upon cell contact. CdiA producing cells protect themselves from auto-inhibition with CdiI immunity proteins, which specifically neutralize toxin activity. Here, we describe a new family of CdiA toxins that block energy production in target bacteria by dissipating the transmembrane electrical potential. Although these toxins share the same activity, they are highly variable in sequence and appear to enter target-cell membranes using different molecular pathways. The associated CdiI proteins exhibit even greater sequence polymorphism, and each immunity protein only protects against its corresponding toxin. The specificity of this toxin-immunity interaction network enables CdiA expressing cells to discern whether neighboring bacteria are genetically identical or nonidentical. This mechanism of self/nonself discrimination can dictate whether bacterial populations engage in antagonistic or collaborative interactions.

## Introduction

Research over the past 20 years shows that bacteria deploy a number of specialized secretion systems to deliver protein toxins directly into competitors [1]. This phenomenon was first described in *Escherichia coli* isolate EC93 as contact-dependent growth inhibition (CDI) [2]. *E*. *coli* EC93 cells use CdiB and CdiA two-partner secretion (TPS) proteins to intoxicate target bacteria [2,3]. CdiB is an outer-membrane β-barrel transporter that exports the toxic CdiA effector [2,4]. CdiA is a large, multi-domain protein that forms an elongated filament projecting from the cell surface [5]. Upon binding a specific receptor, CdiA transfers its toxic C-terminal region (CdiA-CT) into target bacteria to inhibit cell growth [6–10]. Because CdiA-CT toxins are also delivered into neighboring siblings, *E*. *coli* EC93 cells produce a CdiI immunity protein to neutralize CdiA-CT activity and prevent self-intoxication. Related CdiB-CdiA pairs are distributed widely across α-, β- and γ-proteobacteria and are particularly common in human, plant and arthropod pathogens [11–13]. To date, CDI activity has been demonstrated in *E*. *coli* [2,14], *Dickeya dadantii* [11], *Burkholderia thailandensis* [15,16], *Neisseria meningitidis* [17], *Enterobacter cloacae* [18], *Pseudomonas aeruginosa* [19–21], *Acinetobacter baumannii* [22], *Acinetobacter baylyi* [23], *Burkholderia dolosa* [24], and *Burkholderia multivorans* [25]. CDI toxins are remarkably variable in sequence between bacteria, and strains of the same species commonly deploy toxins with different activities. In the *Neisseria* and γ-proteobacteria, the variable CdiA-CT region is usually demarcated by a conserved VENN peptide sequence [11]. CDI toxin diversity is mirrored by CdiI immunity proteins, which necessarily bind cognate CdiA-CTs to block toxic activities. These observations suggest that CDI systems play a role in competition for growth niches and other environmental resources. However, CdiA proteins also function as adhesins and have been shown to foster collaboration between isogenic, immune cells by promoting auto-aggregation and biofilm formation [26–30]. Thus, the polymorphic network of CdiA-CT•CdiI interactions contributes to self/nonself discrimination between bacterial populations.

**Fig 1:**
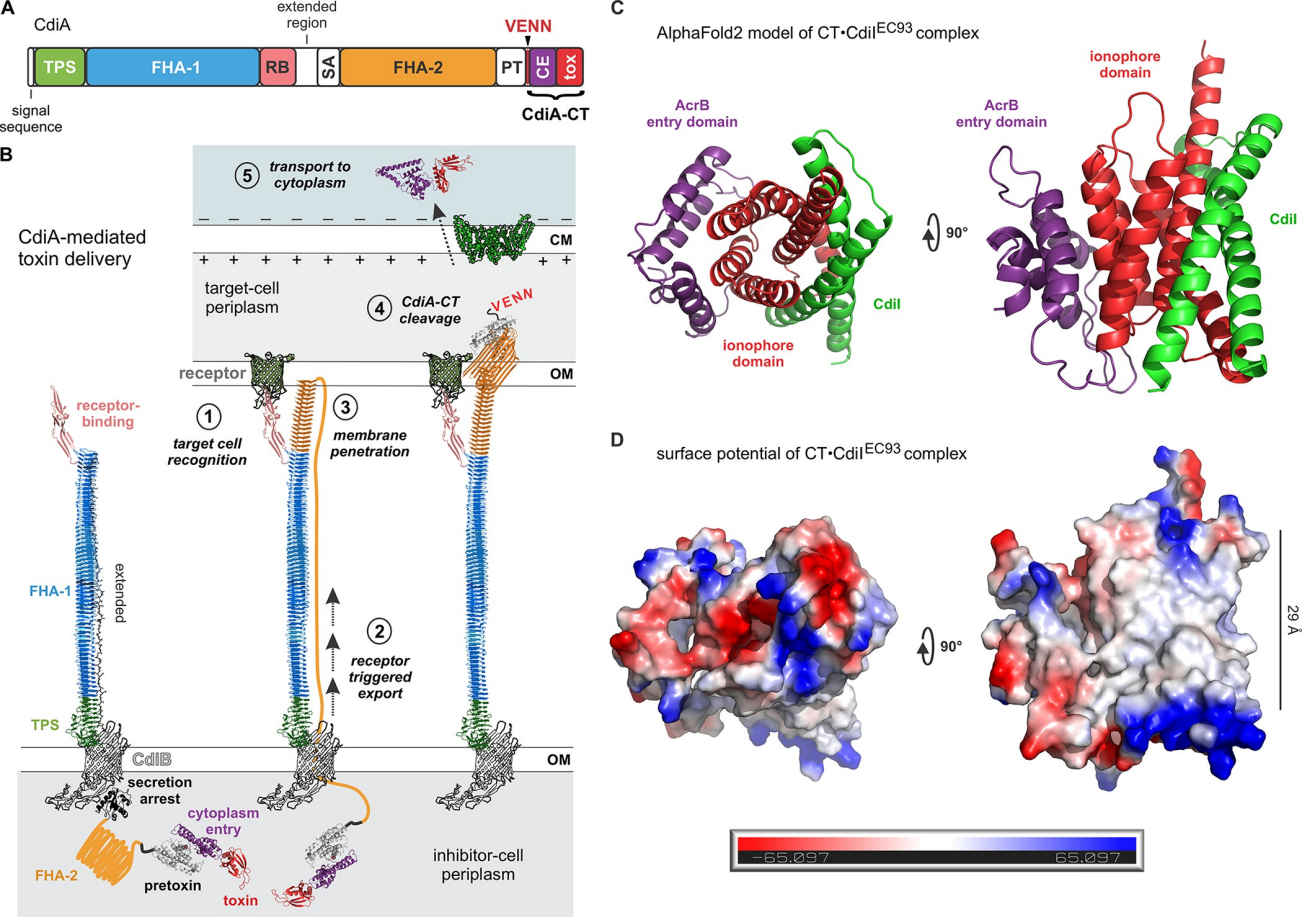
CdiA domain architecture and toxin delivery. **A**) Domain architecture of CdiA. Abbreviations are described in panel B. The VENN peptide motif demarcates the toxic CdiA-CT region, which is cleaved from the effector and released into the target bacterium. **B**) Model for CDI toxin delivery. **C**) AlphaFold2 multimer model of the CT•CdiI^EC93^ complex. **D**) Predicted electrostatic surface potential of the CT•CdiI^EC93^ complex. Structural views are identical in panels C and D.

CDI toxin delivery is a complex multi-step process that has been characterized most extensively in *E*. *coli* [5]. Although CdiA effectors vary in size and sequence between species, they all share the same overall domain architecture (**Fig 1A**) [12,31], suggesting that the mechanism of CdiA-CT delivery is similar across phyla. The domains of CdiA are arranged from N- to C-terminus in the same order that they act during toxin delivery (**Fig 1A** and **1B**). The N-terminal region of CdiA is required for secretion across the cell envelope of the inhibitor cell. A signal peptide first directs CdiA to the periplasm through the Sec machinery, then the TPS transport domain interacts with CdiB to initiate export across the outer membrane [4]. Based on mechanistic studies with homologous FhaB and FhaC TPS proteins from *Bordetella* species, CdiA is thought to be transported through the lumen of CdiB as an unfolded chain [4,32,33]. As the effector emerges from the cell, its filamentous hemagglutinin-1 (FHA-1) peptide repeat domain folds into a right-handed β-helix (**Fig 1B**) [34]. Folding of the β-helix likely provides the driving force for CdiA export, and the resulting extracellular filament projects the receptor-binding domain (RBD) several hundred angstroms from the cell surface (**Fig 1B**) [5]. CdiB-mediated secretion is halted by a conserved α-helical secretion-arrest domain located between the RBD and a second FHA-2 peptide repeat domain in CdiA (**Fig 1A** and **1B**). This export arrest imparts an unusual surface topology to CdiA, with its N-terminal half forming the extracellular filament, while the C-terminal half is sequestered within the periplasm (**Fig 1B**) [5]. CdiA remains in this partially exported state until it engages its receptor, which then triggers secretion to resume (**Fig 1B**, steps 1 & 2). AlphaFold2 modeling suggests that the FHA-2 domain forms a coaxial β-helix extending from the tip of the FHA-1 filament (**Fig 1B**, step 3) [35]. AlphaFold2 also predicts that the FHA-2 domain has extended β-hairpins that project from the central β-helix. Because FHA-2 is required for toxin translocation into the target-cell periplasm [5], the domain presumably forms a transport conduit across the outer membrane (**Fig 1B**). Transfer into the target-cell periplasm then induces autoproteolytic cleavage at the VENN motif to release a CdiA-CT fragment (**Fig 1B**, step 4). The CdiA-CT region is composed of two domains: an N-terminal cytoplasm-entry domain required for translocation across the target-cell inner membrane, and a C-terminal toxin domain with growth inhibition activity (**Fig 1A**) [36]. There are many classes of cytoplasm-entry domains that hijack different integral membrane proteins to mediate translocation to the cytosol [21,36–38]. The import mechanism remains poorly understood, but CDI toxin entry clearly depends on the transmembrane proton gradient and proteolytic processing at the conserved VENN motif [39,40].

Although the majority of characterized CDI toxins are nucleases [11,15,18,21,41–47], the originally discovered CT^EC93^ toxin from *E*. *coli* EC93 dissipates the proton gradient in target cells [2,3]. Loss of the transmembrane electrochemical potential significantly reduces ATP production and inhibits cell growth. This activity suggests that the CT^EC93^ toxin integrates into the cytoplasmic membrane, where it conducts ions down their electrochemical gradients. Accordingly, the cognate CdiI^EC93^ immunity protein is very hydrophobic and predicted to be membrane-localized [3]. AlphaFold2 modeling indicates that the CT^EC93^ toxin is α-helical and interacts with CdiI^EC93^ through its C-terminal domain (**Fig 1C**). The circumference of the CT•CdiI^EC93^ helical bundle is non-polar and thick enough to span the hydrophobic interior of the cell membrane (**Fig 1D**). Genetic studies show that CT^EC93^ toxicity depends on AcrB [6], which is a trimeric inner-membrane protein that functions as a multi-drug efflux pump [48]. How AcrB facilitates intoxication is largely unexplored, but it is presumably exploited as a receptor to promote CT^EC93^ integration into the cytoplasmic membrane. More recently, we identified another depolarizing CDI toxin from *E*. *coli* strain EC869 [49]. This latter CT_o10_^EC869^ toxin shares ~30% sequence identity with CT^EC93^ and depends on SecY as a receptor [49]. These observations suggest that CdiA effectors carry various ionophores that exploit distinct pathways to assemble into the target-cell membrane.

Here, we perform a systematic search for CT^EC93^ homologs and identify distantly related toxins across the β- and γ-proteobacteria. AlphaFold2 modeling indicates that these toxins share a common C-terminal five-helix bundle that likely constitutes the ionophore domain. By contrast, the predicted N-terminal entry domains vary in structure, suggesting that they recognize different receptors. Ionophoric CdiA-CT toxins from *E*. *coli* isolates have radiated into six major groups defined by their entry domain structures. Using comparative sequence analysis and forward genetics, we show that *E*. *coli* ionophores utilize AcrB (groups 1 & 3), SecY (group 2), YciB (group 4), PuuP/PlaP (group 5) and DtpA/DtpB (group 6) as inner membrane receptors to intoxicate target bacteria. Our analyses also reveal striking intra-group sequence variation in helices α1, α4 and α5 of the ionophore domains. These variable toxin residues are predicted to interact directly with CdiI immunity proteins, which are also highly polymorphic. Competition co-culture experiments confirm that CdiI proteins only protect against their cognate ionophores. These findings demonstrate that the CDI ionophore-immunity protein family has diversified into a large number of distinct cognate pairs.

## Results

### Identification of new CDI ionophore toxins

**Fig 2:**
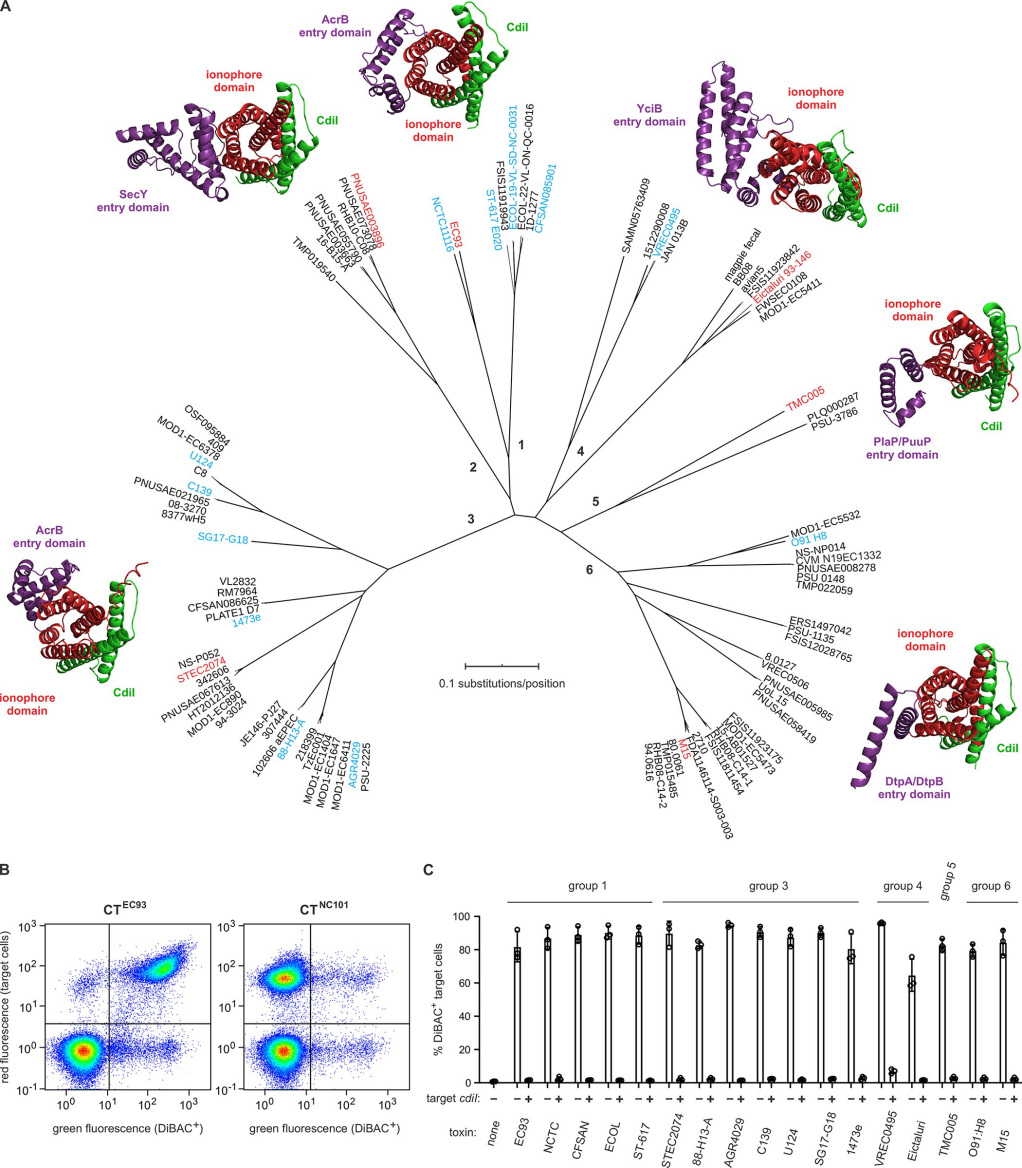
CDI ionophore toxins from *E*. *coli* strains. **A**) Predicted ionophore toxins from *E*. *coli* isolates. Six major groups of CDI ionophores were identified using iterative PSI-BLAST searches with the CdiA-CT^EC93^ sequence as a query. NCBI reference sequence numbers for the CdiA and CdiI proteins are provided in **S1 Table**. AlphaFold2 models are presented for representative CT•CdiI complexes from the strains highlighted in red font. CdiA-CT entry and ionophore domains are colored violet and red, respectively. CdiI proteins are rendered in green. The ionophore domains of each toxin were superimposed, and the structures are viewed down the long axis of the five-helix bundle. CT•CdiI pairs from the strains highlighted in cyan were tested for ionophore activity in panel C. **B**) Flow cytometric assay for ionophore activity. Red fluorescent *E*. *coli* target bacteria were incubated with unlabeled inhibitor cells that deploy either the CT^EC93^ ionophore or CT^NC101^ RNase. Cell suspensions were then treated with DiBAC4(3) to stain depolarized bacteria for flow cytometry. Depolarized target cells were quantified as dual fluorescent events in the upper right quadrant. **C**) Quantification of target cell depolarization. Red fluorescent *E*. *coli* target bacteria were incubated with inhibitor strains that deploy the indicated toxins from each ionophore group. Where indicated (+) the target cells express the cognate *cdiI* immunity gene. The percentage of depolarized target cells was quantified by flow cytometry. Presented data are the average ± standard error for three independent experiments.

CdiA^EC93^ was shown to disrupt the transmembrane potential some 15 years ago [3,11], yet the ionophoric CT^EC93^ toxin still has no designation in current databases and its phylogenetic distribution remains unexplored. We queried the NCBI protein database with the CT^EC93^ sequence (excluding the conserved VENN motif) and recovered hundreds of putative CdiA effector proteins from Enterobacteriacae, Pseudomonadales, Oceanspirillales, Neisseriales and Burkholderiales. We then used iterative PSI-BLAST searches to identify all related ionophoric CdiA proteins from *E*. *coli* isolates. The latter searches yielded 864 effectors that carry 89 unique CT sequences (**Fig 2A**, **S1 Table** and **S1 Appendix**). These toxins are very diverse, and the only shared sequence element is a putative glycine-zipper motif corresponding to residues Gly168-X-X-X-Gly172-X-X-X-Gly176 in CT^EC93^ (**S1 Fig**). Though heterogeneous in sequence, AlphaFold2 predicts that each toxin is composed of two α-helical domains (**Figs 2A** and **S2** and **S2 Appendix**). This bipartite organization is similar to previously characterized CDI toxins, which contain N-terminal cytoplasm-entry domains linked to C-terminal nucleases [36,40,50]. By analogy with CdiA-CT nuclease toxins, we hypothesize that the N-terminal entry domains (**Fig 2A**, in violet) act to insert the C-terminal ionophore domains (**Fig 2A**, in red) into target-cell membranes. The *E*. *coli* CDI toxins partition into six major groups based on the predicted structures of their entry domains (**Figs 2A** and **S1**). The putative CdiI immunity proteins for these toxins are all small (72–100 residues) and very hydrophobic (**S1 Table** and **Fig 2A**, in green). Membrane topology predictions indicate that the immunity proteins form two-helix hairpins with N- and C-termini located in the cytoplasm. AlphaFold2 multimer modeling predicts that each CdiI protein interacts with helices α1, α4 and α5 of its cognate ionophore domain (**Figs 2A** and **S2**and **S2 Appendix**).

To test the newly identified toxins for ionophore activity, we generated chimeric CdiA proteins that deliver representative CTs and used a previously described flow cytometry method to monitor target-cell depolarization [49]. Inhibitor cells were first mixed at a 1:1 ratio with dTomato-labeled target bacteria for 1 h to allow toxin delivery. The co-cultures were then incubated with bis-(1,3-dibutylbarbituric acid)-trimethine oxonol [DiBAC_4_(3)], which is an anionic dye that only fluoresces upon entering depolarized cells [51]. Approximately 82% of target cells exhibit DiBAC_4_(3) fluorescence when co-cultured with inhibitor cells that deploy CT^EC93^ (**Fig 2B**). By contrast, only ~2% of target bacteria show DiBAC_4_(3) fluorescence when intoxicated with a CDI tRNase toxin from *E*. *coli* NC101 (**Fig 2B**) [44]. The latter result shows that DiBAC_4_(3) staining can discriminate depolarized cells from bacteria killed by other toxic activities. Analysis of representatives from each ionophore group revealed that all of the toxins depolarize target cells (**Fig 2C**). Moreover, target bacteria are protected from intoxication/depolarization when they express cognate *cdiI* immunity genes (**Fig 2C**). Together, these results indicate that *E*. *coli* strains collectively encode a large family of CDI ionophore toxins.

### Group 1 ionophores use AcrB to intoxicate target cells

**Fig 3:**
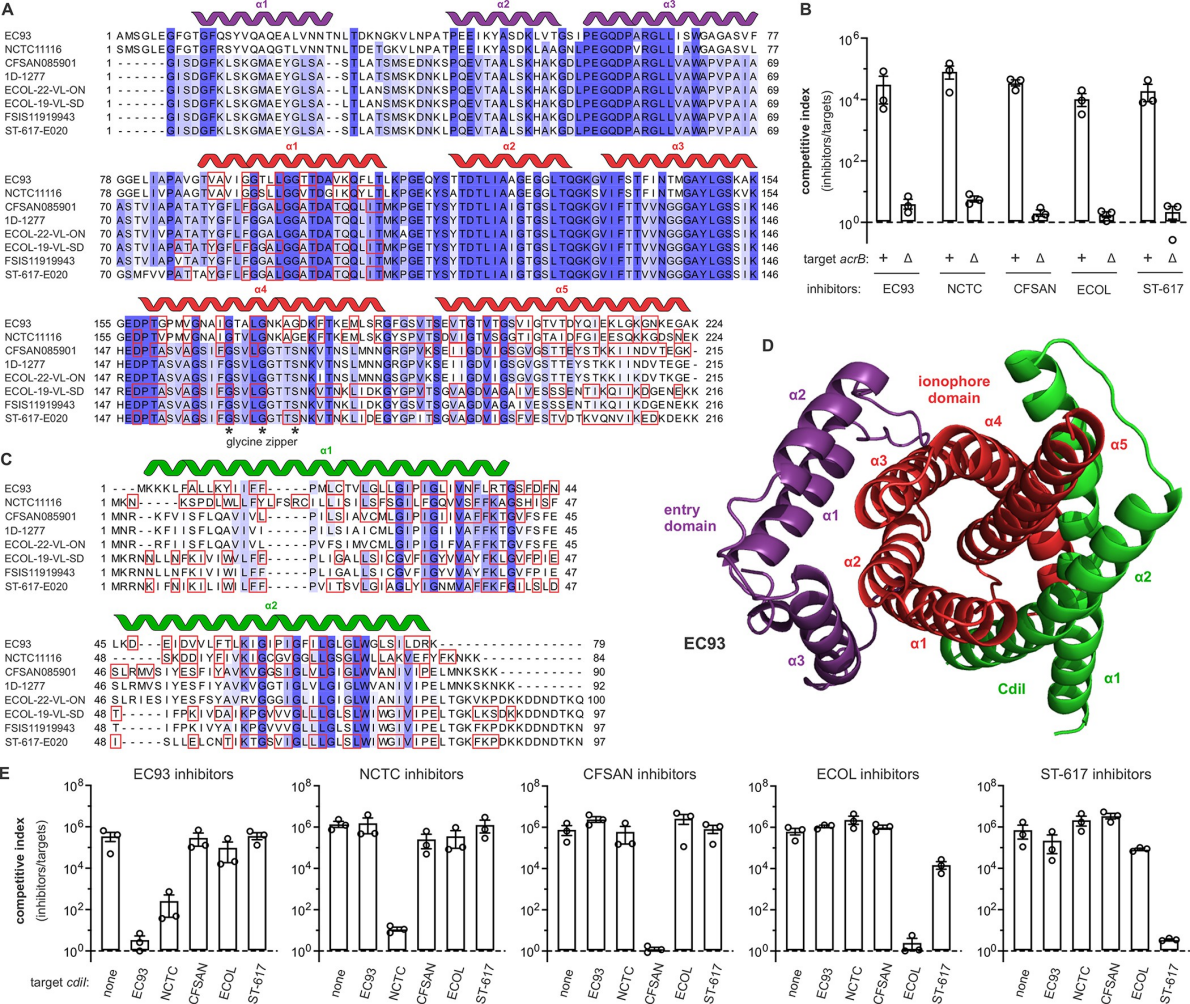
Group 1 AcrB-dependent CDI ionophore toxins. **A**) Alignment of unique group 1 ionophore toxins from *E*. *coli* isolates. Predicted α-helical secondary structure elements for entry and ionophore domains are depicted above the alignment in violet and red, respectively. AlphaFold2 predicts that the residues in red boxes make direct contact with CdiI immunity proteins. The putative glycine zipper motif in helix α4 is indicated by asterisks (*). **B**) Group 1 ionophores are AcrB-dependent. Inhibitor strains that deploy the indicated toxins were co-cultured with *acrB*^*+*^ and Δ*acrB* target bacteria at a 1:1 ratio for 3 h. Viable inhibitor and target cells were enumerated and competitive indices calculated as the final ratio of inhibitor to target bacteria divided by the initial ratio. Presented data are the average ± standard error for three independent experiments. **C**) Alignment of unique group 1 CdiI immunity proteins from *E*. *coli* isolates. Predicted α-helical secondary structure elements are depicted above the alignment. AlphaFold2 predicts that the residues in red boxes make direct contact with CdiA- CT ionophore domains. **D**) AlphaFold2 model of the CT•CdiI^EC93^ complex. The proteins are color-coded and secondary structure elements labeled according to panels A and C. **E**) Specificity of group 1 CT•CdiI interactions. Inhibitor strains that deploy the indicated toxins were co-cultured with target bacteria that express the indicated *cdiI* immunity genes at a 1:1 ratio for 3 h. Viable inhibitor and target cells were enumerated and competitive indices calculated as the final ratio of inhibitor to target bacteria divided by the initial ratio. Presented data are the average ± standard error for three independent experiments.

The group 1 ionophores include eight unique CdiA-CT sequences that are most closely related to CT^EC93^ (**Fig 3A**). Members of this group are distributed across 40 isolates of *E*. *coli*, though only one toxin from *E*. *coli* NCTC:11116 (CT^NCTC^) shares greater that 70% sequence identity with CT^EC93^ (**Figs 2A** and **3A**). The other group 1 toxins are ~40% identical to CT^EC93^/CT^NCTC^ and have entry domains with distinct sequences (**Fig 3A**), suggesting that they may use a different membrane-insertion pathway. Previous work indicates that CT^EC93^ exploits AcrB as an inner membrane receptor to intoxicate target cells [6]. Accordingly, we found that Δ*acrB* target cells are resistant to the CT^NCTC^ toxin (**Fig 3B**), which shares a nearly identical entry domain with CT^EC93^ (**Fig 3A**). Somewhat surprisingly, other group 1 toxins from *E*. *coli* CFSAN085901 (CT^CFSAN^), *E*. *coli* ECOL-19-VL-SD-NC-0031 (CT^ECOL^) and *E*. *coli* ST-617_E020 (CT^ST-617^) are also AcrB-dependent (**Fig 3B**). Given the sequence divergence between group 1 entry domains, these results suggest that the subgroups recognize different regions on AcrB. Alternatively, the common PEGQDP(A/V)RGLL sequence motif in helix α3 of all group 1 entry domains may mediate the interaction with AcrB (**Fig 3A**).

### Ionophore toxins and immunity proteins have evolved into diverse cognate pairs

Group 1 toxins also exhibit sequence polymorphism concentrated in helices α1, α4 and α5 of the ionophore domain (**Fig 3A**). These α-helices are predicted to interact directly with CdiI (**Fig 3A**, **3C**, **3D** and **S2 Appendix**), suggesting that each immunity protein may only protect against its cognate toxin. To test the specificity of group 1 CT•CdiI interactions, we competed each CDI^+^ inhibitor strain against a panel of target bacteria that express the various immunity genes. As expected, target cells are protected when they express cognate *cdiI*, but remain susceptible to other ionophore toxins (**Fig 3E**). For example, bacteria expressing *cdiI*^CFSAN^ are fully resistant to CT^CFSAN^ intoxication, but are inhibited by other group 1 ionophores to the same extent as target cells that carry no immunity gene at all (**Fig 3E**). In some instances, we observed partial cross-resistance between toxin-immunity pairs. *cdiI*^NCTC^ expression confers a ~100-fold fitness benefit against CT^EC93^ inhibitors compared to target bacteria that express other non-cognate immunity genes (**Fig 3E**). This result is consistent with the ~78% sequence identity shared by CT^EC93^ and CT^NCTC^ (**Fig 3A**). We note that CdiI^EC93^ and CdiI^NCTC^ are only 36% identical (**Fig 3C**), which may explain the lack of reciprocal cross-protection when *cdiI*^EC93^ expressing target bacteria are co-cultured with CT^NCTC^ inhibitor cells (**Fig 3E**). In addition, partial immunity to CT^ECOL^ intoxication was observed with *cdiI*^ST-617^ expressing target cells. The CdiI^ECOL^ and CdiI^ST-617^ proteins share ~60% identity, but differ at several positions that are predicted to contact the ionophore domain (**Fig 3C**). Together, these observations indicate that group 1 ionophores and immunity proteins have diverged into distinct cognate pairs.

**Fig 4:**
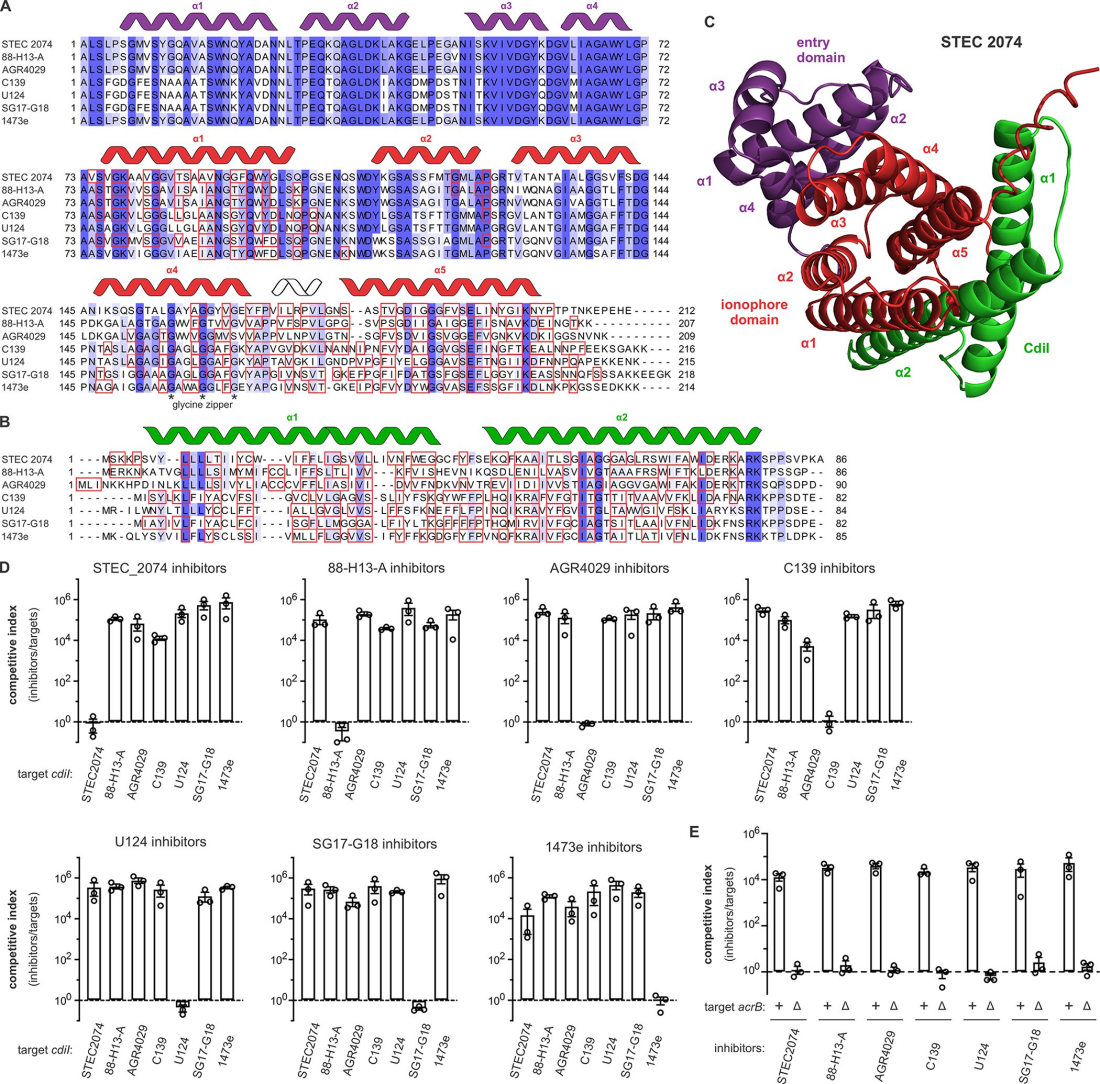
Group 3 AcrB-dependent CDI ionophore toxins. **A**) Alignment of representative group 3 ionophore toxins from *E*. *coli* isolates. Predicted α-helical secondary structure elements for entry and ionophore domains are depicted above the alignment in violet and red, respectively. AlphaFold2 predicts that the residues in red boxes make direct contact with CdiI immunity proteins. **B**) Alignment of representative group 3 CdiI immunity proteins from *E*. *coli* isolates. Predicted α-helical secondary structure elements are depicted above the alignment. AlphaFold2 predicts that the residues in red boxes make direct contact with CdiA-CT ionophore domains. **C**) AlphaFold2 model of the CT•CdiI^STEC2074^ complex. The proteins are color-coded and secondary structure elements labeled according to panels A and B. **D**) Specificity of group 3 CT•CdiI interactions. Inhibitor strains that deploy the indicated toxins were co-cultured with target bacteria that express the indicated *cdiI* immunity genes at a 1:1 ratio for 3 h. Viable inhibitor and target cells were enumerated and competitive indices calculated as the final ratio of inhibitor to target bacteria divided by the initial ratio. Presented data are the average ± standard error for three independent experiments. **E**) Group 3 ionophores are AcrB-dependent. Inhibitor strains that deploy the indicated toxins were co-cultured with *acrB*^*+*^ and Δ*acrB* target bacteria at a 1:1 ratio for 3 h. Viable inhibitor and target cells were enumerated and competitive indices calculated as the final ratio of inhibitor to target bacteria divided by the initial ratio. Presented data are the average ± standard error for three independent experiments.

Intra-group sequence polymorphism is also a prominent feature of the other *E*. *coli* ionophore-immunity protein pairs (**S3**, **S4**, **S5**, **S6**and **S7 Figs**). For example, group 3 toxins ramify into seven sub-groups (**Figs 2A, 4A** and **S4A**). This clustering is mirrored by the group 3 immunity proteins (**Figs 4B** and **S4B**), with most of the variable positions mapping to the predicted CT•CdiI binding interface (**Fig 4C** and **S2 Appendix**). To determine whether these sub-groups represent distinct cognate pairs, we competed inhibitor cells that deploy toxins from *E*. *coli* strains STEC 2074 (CT^STEC2074^), 88-H13-A (CT^88-H13-A^), AGR4029 (CT^AGR4029^), C139 (CT^C139^), U124 (CT^U124^), SG17-G18 (CT^SG17^) and 1473e (CT^1473e^) against a panel of target bacteria that express the corresponding *cdiI* immunity genes. These analyses show that only cognate immunity proteins are able to protect target bacteria against intoxication (**Fig 4D**). Given the specificity observed with representative group 1 and 3 toxins, we predict that *E*. *coli* isolates encode ~30 distinct ionophore-immunity protein pairs (**Fig 2A**).

### Group 3 and 4 ionophores recognize previously identified CdiA-CT receptors

**Fig 5:**
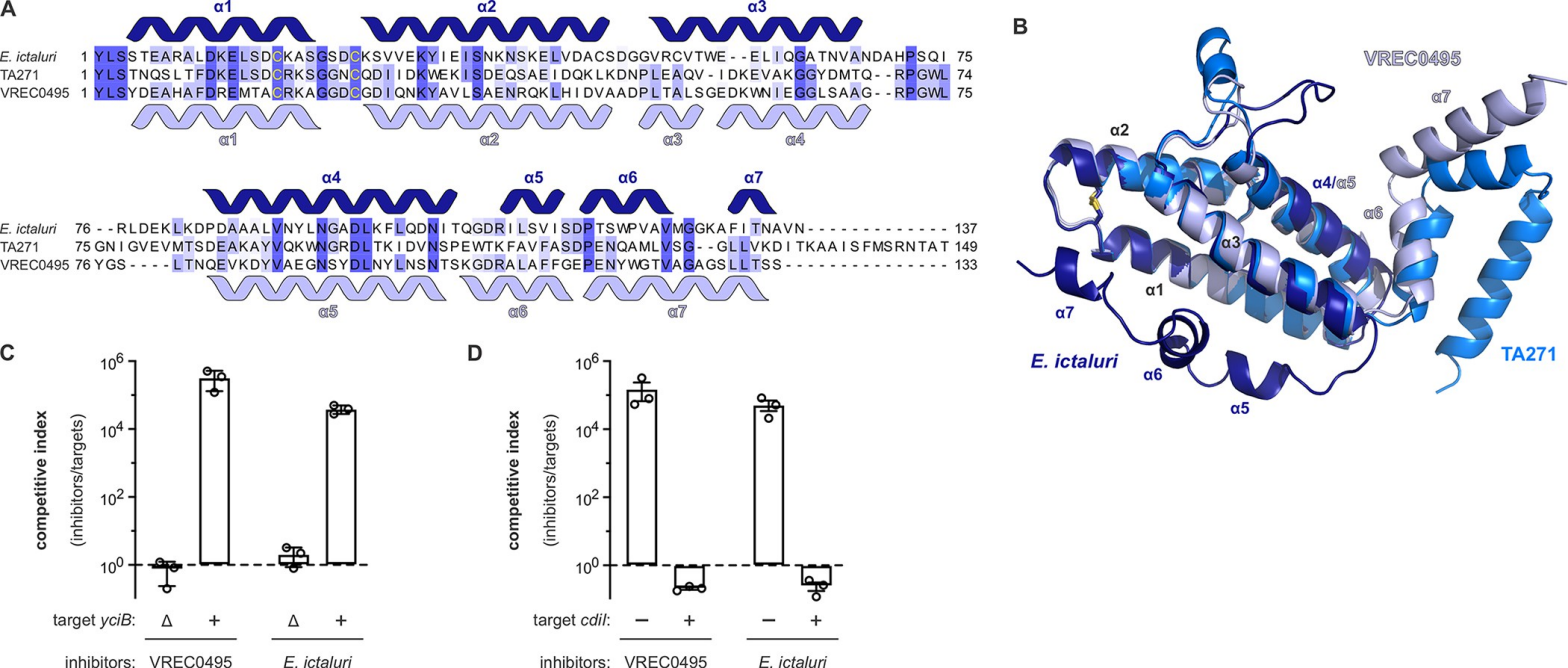
Group 4 CDI ionophore toxins utilize YciB as a receptor. **A**) Alignment of the entry domain of the YciB-dependent entry domain from CT^TA271^ with the predicted entry domains of CT^VREC0495^ and CT^Eict^ toxins. Predicted α-helical secondary structure elements for CT^Eict^ and CT^VREC0495^ are indicated above and below (respectively) the alignment. Cys residues predicted to form a disulfide bond are indicated in yellow. **B**) Superimposition of predicted entry domain structures from CT^TA271^, CT^VREC0495^ and CT^Eict^. The predicted disulfide linking α1 and α2 is rendered as sticks. **C**) Group 4 ionophores are YciB-dependent. Inhibitor strains that deploy the indicated toxins were co-cultured with *yciB*^*+*^ and Δ*yciB* target bacteria at a 1:1 ratio for 3 h. Viable inhibitor and target cells were enumerated and competitive indices calculated as the final ratio of inhibitor to target bacteria divided by the initial ratio. Presented data are the average ± standard error for three independent experiments. **D**) CdiI immunity protein protects target cells from group 4 ionophore intoxication. Inhibitor strains that deploy the indicated toxins were co-cultured with target bacteria that express the indicated *cdiI* immunity genes at a 1:1 ratio for 3 h. Viable inhibitor and target cells were enumerated and competitive indices calculated as the final ratio of inhibitor to target bacteria divided by the initial ratio. Presented data are the average ± standard error for three independent experiments.

Our previous work indicates that group 2 ionophores use SecY as an inner membrane receptor [49], but the remaining toxin groups have not been characterized. PSI-BLAST revealed homology between the entry domains of AcrB-dependent Ntox25 toxins and group 3 ionophores (**S1 Appendix**) [14], and accordingly we found that Δ*acrB* mutants are resistant to all representative group 3 toxins (**Fig 4E**). Homology searches and AlphaFold2 predictions also suggest that group 4 entry domains are similar to the YciB-dependent entry domain of a previously characterized CDI DNase toxin from *E*. *coli* TA271 (**Fig 5A and 5B** and **S1 Appendix**) [36,41,52]. Therefore, we co-cultured Δ*yciB* mutants with inhibitor strains that deploy group 4 toxins from *E*. *coli* VREC0495 (CT^VREC0495^) and *Edwardsiella ictaluri* (CT^Eict^) and found that the target bacteria are resistant to both toxins (**Fig 5C**). Notably, this resistance phenotype is comparable to that exhibited by *yciB*^*+*^ cells that express cognate immunity genes (**Fig 5D**).

### Group 5 toxins hijack paralogous putrescine importers

**Fig 6:**
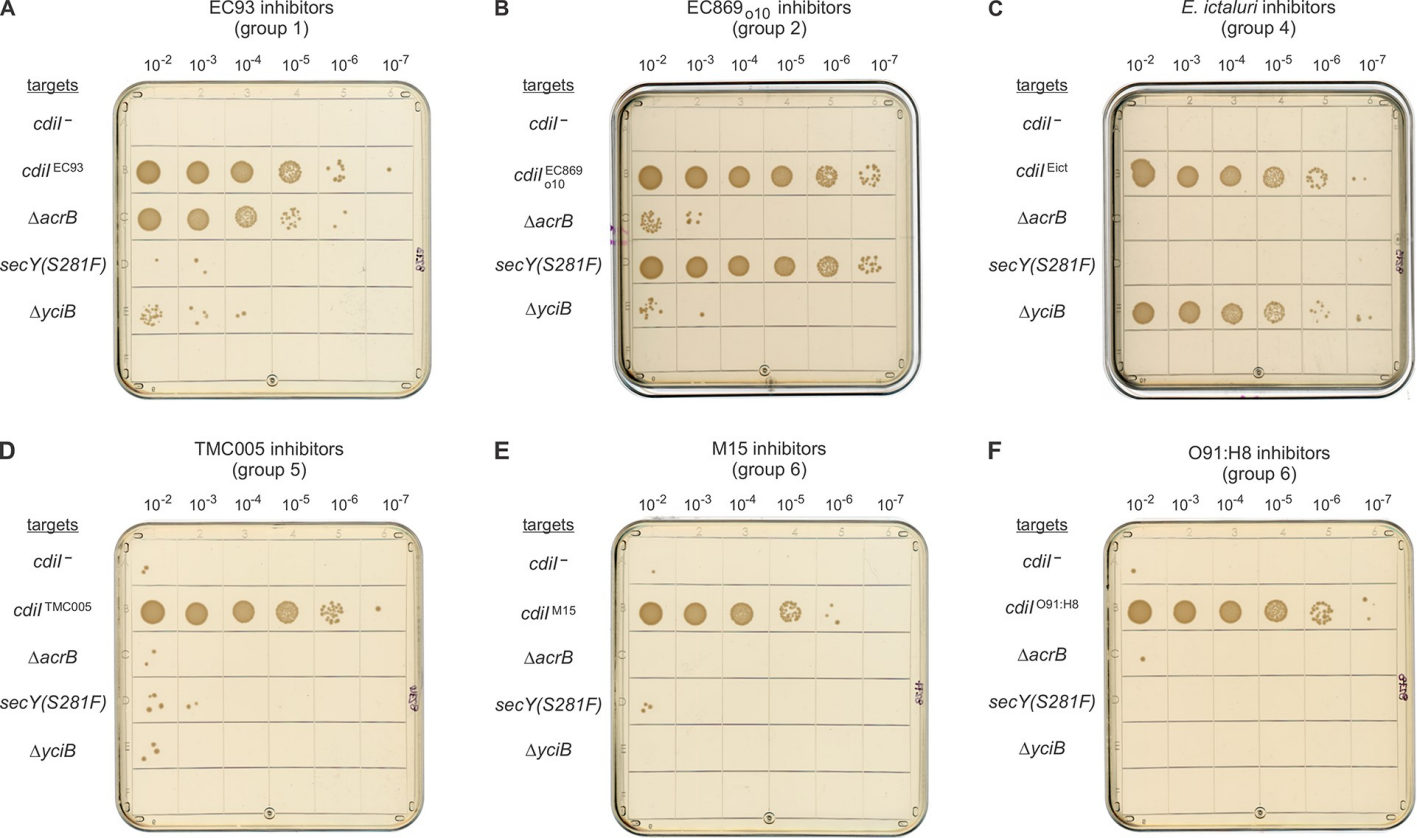
Group 5 and 6 ionophore toxins exploit novel receptors. **A**) Inhibitor cells that deploy CT^EC93^ were co-cultured at a 1:1 ratio with the indicated target bacteria for 3 h, and 10-fold serial dilutions were plated onto Spc-supplemented medium to enumerate viable target cells. The same procedure was followed with inhibitor strains that deploy CT_o10_^EC869^ (**B**), CT^Eict^ (**C**), CT^TMC005^ (**D**), CT^M15^ (**E**) and CT^O91:H8^ (**F**).

Given that divergent entry domains are able to utilize the same receptor, we asked whether the uncharacterized group 5 and 6 entry domains recognize known receptors. We first confirmed that Δ*acrB*, *secY(Ser281Phe)* and Δ*yciB* target strains are specifically resistant to group 1 (**Fig 6A**), group 2 (**Fig 6B**) and group 4 (**Fig 6C**) ionophores, respectively. In each instance, the receptor mutations provide protection similar to that afforded by the cognate immunity protein (**Fig 6A**, **6B** and **6C**). We then challenged the target strains with inhibitors that deploy group 5 CT^TMC005^ and found that all were inhibited to the same extent as wild-type cells (**Fig 6D**). Similarly, group 6 toxins from *E*. *coli* strains M15 (CT^M15^) and O91:H8 (CT^O91:H8^) inhibit the growth of Δ*acrB*, *secY(Ser281Phe)* and Δ*yciB* target cells (**Fig 6E** and **6F**). Together, these data indicate that group 5 and 6 ionophores recognize novel membrane protein receptors.

**Fig 7:**
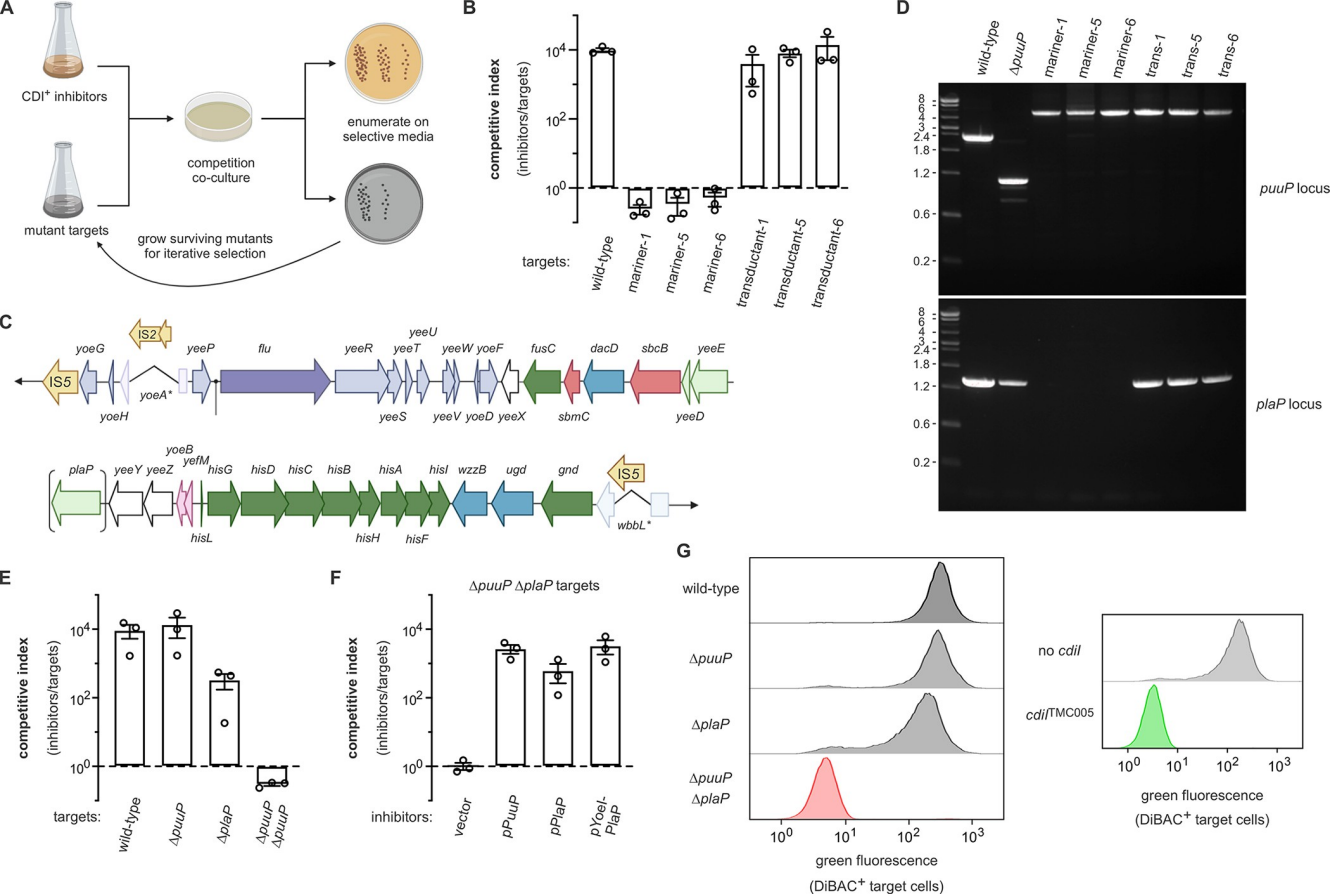
Group 5 ionophore toxins utilize paralogous putrescine import proteins as receptors. **A**) Selection scheme to isolate CDI-resistant *mariner* transposon insertion mutants. **B**) Transposon insertions in *puuP* were identified in selections for CT^TMC005^ resistant mutants, but *puuP* disruptions are not sufficient to confer resistance. Inhibitor strains that deploy CT^TMC005^ were co-cultured at a 1:1 ratio with the indicated target bacteria for 3 h. Viable inhibitor and target cells were enumerated and competitive indices calculated as the final ratio of inhibitor to target bacteria divided by the initial ratio. Presented data are the average ± standard error for three independent experiments. **C**) Genomic region deleted in CT^TMC005^ resistant mutants. **D**) PCR analyses of the *puuP* and *plaP* loci from CT^TMC005^ resistant *mariner* mutants. **E**) CT^TMC005^ exploits the paralogous putrescine transporters PuuP and PlaP as receptors. Inhibitor strains that deploy CT^TMC005^ were co-cultured at a 1:1 ratio with the indicated target bacteria for 3 h. Viable inhibitor and target cells were enumerated and competitive indices calculated as the final ratio of inhibitor to target bacteria divided by the initial ratio. Presented data are the average ± standard error for three independent experiments. **F**) Complementation of Δ*puuP* Δ*plaP* mutants restores sensitivity to CT^TMC005^ intoxication. Inhibitor strains that deploy CT^TMC005^ were co-cultured at a 1:1 ratio with Δ*puuP* Δ*plaP* target bacteria that carry the indicated plasmids for 3 h. Viable inhibitor and target cells were enumerated and competitive indices calculated as the final ratio of inhibitor to target bacteria divided by the initial ratio. Presented data are the average ± standard error for three independent experiments. **G**) Δ*puuP* Δ*plaP* mutants are not depolarized by CT^TMC005^. The indicated red fluorescent target strains were incubated with inhibitors that deploy CT^TMC005^ for 1 h, then the cell suspensions were treated with DiBAC4(3) to label depolarized cells for quantification by flow cytometry. The protection afforded by the *cdiI*^TMC005^ immunity gene is also presented for comparison. Panels A and C were created in BioRender. Halvorsen, T. (2020) https://BioRender.com/o68v649.

To identify the receptors for group 5 and 6 toxins, we selected for CDI-resistant mutants, reasoning that disruption of the receptor-encoding genes should protect against intoxication. Six independent pools of *mariner* transposon mutants were prepared and co-cultured with CT^TMC005^ and CT^M15^ inhibitor strains to select for resistant clones (**Fig 7A**). After three iterative rounds of selection, we obtained CT^TMC005^ resistant clones from three of the *mariner* pools (**Fig 7B**), but failed to isolate any CT^M15^ resistant mutants. The CT^TMC005^ resistant mutants carry independent transposon insertions in *puuP*, which encodes a high-affinity putrescine import protein [53,54]. However, these *puuP* disruptions do not confer resistance when transferred into wild-type *E*. *coli* cells by phage P1 mediated transduction (**Fig 7B**), suggesting that the original clones contain additional unidentified mutations that contribute to resistance.

Given that transposon insertions typically generate null alleles, it is possible that this mutagenesis approach failed to yield CDI-resistant mutants because the sought-after receptor proteins are essential for viability. Therefore, ultraviolet (UV) irradiation was used to induce a diverse spectrum of mutations for the selections. Because substitutions in the extracellular loops of BamA confer resistance to the CdiA^EC93^ effector used here for toxin delivery [9,55], we also provided the target cells with plasmid-borne *bamA* to avoid selecting mutations in this gene. CT^TMC005^ resistant clones were isolated from all six of the UV-irradiated mutant pools. Whole-genome sequencing revealed *puuP* mutations in all isolates (**Table 1**), again implicating the putrescine transporter in CT^TMC005^ activity. The resistant clones also harbor the same ~34.5 kbp deletion extending from nucleotides 2,064,549 to 2,098,995 in the *E*. *coli* MG1655 genome (**Table 1**and **Fig 7C**). This region is flanked by directly repeated IS*5* elements, suggesting that each deletion arose independently through homologous recombination between these identical sequences (**Fig 7C**). Notably, the deleted segment includes *plaP*, which encodes a low-affinity putrescine importer that shares 63% sequence identity with PuuP (**S8 Fig**) [56]. Together, these observations suggest that PuuP and PlaP provide parallel membrane-entry pathways for CT^TMC005^. Given that *plaP* is absent from all of the UV-generated mutants, we tested whether the initially isolated *puuP* transposon mutants also contain the IS*5* deletion. PCR amplification of the *puuP* locus confirmed that the original mutants carry insertions, but we were unable to amplify *plaP* from any of the *mariner* mutants (**Fig 7D**). Together, these findings strongly suggest that *puuP* and *plaP* must both be disrupted for resistance to CT^TMC005^.

**Table 1:** Mutations identified in CDI-resistant isolates.

selection toxin	UV pool	mutations
CT^TMC005^	1	*puuP(Lys92-fs)* Δ*(yoeG-gnd)*
	1	*puuP*(Δ*Gly249-Ala256*) Δ(*yoeG-gnd*)
	2	*puuP(Ser228amber)* Δ*(yoeG-gnd)*
	3	*puuP(Tyr248amber)* Δ*(yoeG-gnd)*
	4	*puuP(Pro405Leu)* Δ*(yoeG-gnd)*
	5	*puuP(Pro405Leu)* Δ*(yoeG-gnd)*
	6	*puuP(Trp104opal)* Δ*(yoeG-gnd)*
CT^M15^	2	*dtpA(Thr61fs) dtpB(Lys43ochre) dtpC(Trp66amber)*
	5	*dtpA(Glu397opal) dtpB(Val40-fs)*

To determine whether PuuP and PlaP provide redundant entry pathways, we examined Δ*puuP*, Δ*plaP* and Δ*puuP* Δ*plaP* target strains in competition co-cultures with CT^TMC005^ inhibitors. *E*. *coli* Δ*puuP* cells are inhibited to the same extent as wild-type cells, but Δ*plaP* mutants exhibit partial resistance to CT^TMC005^ (**Fig 7E**). As anticipated, target cells deleted for both *puuP* and *plaP* are completely resistant to intoxication (**Fig 7E**). The partial-resistance phenotype of Δ*plaP* mutants suggests that PlaP may be the preferred receptor for CT^TMC005^. However, complementation of Δ*puuP* Δ*plaP* cells with plasmid-borne *plaP* does not restore CDI susceptibility to the same extent as complementation with *puuP* (**Fig 7F**). Inspection of the *plaP* locus reveals that the first two codons of the open-reading frame overlap with the 3´-coding region of the *yoeI* gene, which encodes a 20-residue peptide of unknown function [57]. Because the Δ*plaP* deletion also disrupts *yoeI*, we generated an additional complementation construct that includes both *yoeI* and *plaP*. This latter construct fully restores sensitivity to CT^TMC005^ (**Fig 7F**), suggesting that *yoeI* may affect PlaP expression. We also examined target cell intoxication by flow cytometry and found that Δ*puuP* and Δ*plaP* single mutants are depolarized when co-cultured with CT^TMC005^ inhibitors (**Fig 7G**). By contrast, Δ*puuP* Δ*plaP* double mutants retain the membrane potential, similar to target cells that express the *cdiI*^TMC005^ immunity gene (**Fig 7G**). Thus, the PuuP and PlaP putrescine importers provide parallel toxin entry pathways for group 5 ionophores.

### Group 6 toxins hijack proton-dependent dipeptide importers

**Fig 8:**
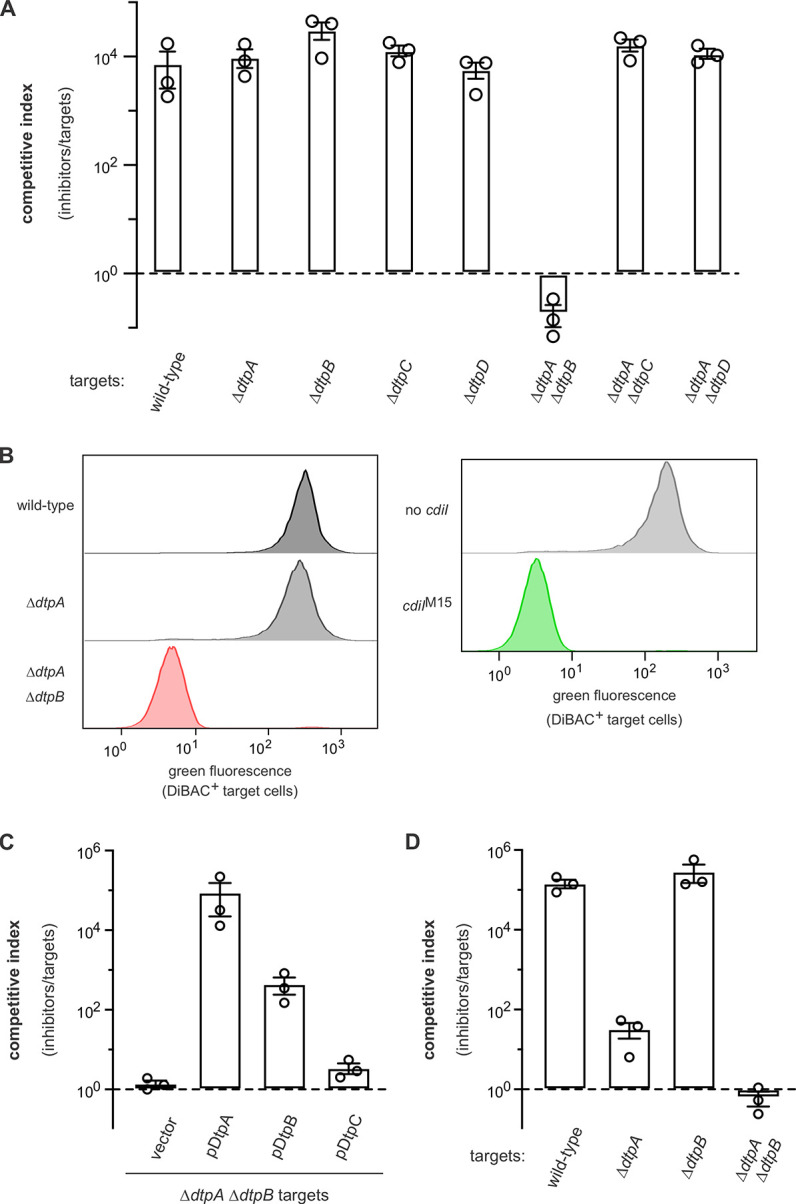
Group 6 ionophore toxins utilize paralogous di/tripeptide import proteins as receptors. **A**) Δ*dtpA* Δ*dtpB* mutants are resistant to intoxication by CT^M15^. Inhibitor strains that deploy CT^M15^ were co-cultured at a 1:1 ratio with the indicated target bacteria for 3 h. Viable inhibitor and target cells were enumerated and competitive indices calculated as the final ratio of inhibitor to target bacteria divided by the initial ratio. Presented data are the average ± standard error for three independent experiments. **B**) Δ*dtpA* Δ*dtpB* mutants are not depolarized by CT^M15^. The indicated red fluorescent target strains were incubated with inhibitors that deploy CT^M15^ for 1 h, then the cell suspensions were treated with DiBAC4(3) to label depolarized cells. DiBAC4(3) fluorescence was quantified by flow cytometry. The protection afforded by the *cdiI*^M15^ immunity gene is also presented for comparison. **C**) Complementation of Δ*dtpA* Δ*dtpB* mutants restores sensitivity to CT^M15^ intoxication. Inhibitor strains that deploy CT^M15^ were co-cultured at a 1:1 ratio with Δ*dtpA* Δ*dtpB* target bacteria that carry the indicated plasmids for 3 h. Viable inhibitor and target cells were enumerated and competitive indices calculated as the final ratio of inhibitor to target bacteria divided by the initial ratio. Presented data are the average ± standard error for three independent experiments. **D**) Δ*dtpA* Δ*dtpB* mutants are resistant to CT^O91:H8^. Inhibitor strains that deploy CT^O91:H8^ were co-cultured at a 1:1 ratio with the indicated target bacteria for 3 h. Viable inhibitor and target cells were enumerated and competitive indices calculated as the final ratio of inhibitor to target bacteria divided by the initial ratio. Presented data are the average ± standard error for three independent experiments.

Although we failed to obtain CT^M15^ resistant *mariner* mutants, two CT^M15^ resistant clones were isolated using the UV mutagenesis approach. Genome sequencing revealed that the pool 2 isolate carries null mutations in *dtpA*, *dtpB* and *dtpC*, and the pool 5 clone harbors mutations in *dtpA* and *dtpB* (**Table 1**). The *dtp* genes encode proton gradient-dependent di/tripeptide transporters, suggesting that CT^M15^ also exploits parallel entry pathways. *E*. *coli* MG1655 contains four of these paralogous transport proteins. DtpA and DtpB are more closely related to each other (52% identity) than to DtpC, which shares 57% sequence identity with DtpD (**S9 Fig**). Target strains carrying individual Δ*dtpA*, Δ*dtpB*, Δ*dtpC* and Δ*dtpD* deletions are susceptible to CT^M15^ intoxication, but Δ*dtpA* Δ*dtpB* double mutants are fully resistant (**Fig 8A**). Accordingly, DiBAC_4_(3) labeling assays indicate that wild-type and Δ*dtpA* target bacteria are depolarized to the same extent upon intoxication with CT^M15^, whereas Δ*dtpA* Δ*dtpB* mutants phenocopy immune target cells that express *cdiI*^M15^ (**Fig 8B**). Δ*dtpA* Δ*dtpB* cells are re-sensitized to CT^M15^ intoxication when provided with plasmid-borne *dtpA*, whereas complementation with *dtpB* is less effective (**Fig 8C**). We also tested Δ*dtpA* Δ*dtpB* target cells that express *dtpC* and found that they remain resistant to CT^M15^ (**Fig 8C**). Together, these results indicate that CT^M15^ can hijack either DtpA or DtpB to gain entry into the cell membrane. We note that some group 6 ionophores–exemplified by CT^O91:H8^ –have distinct entry domain sequences (**Figs 2A** and **S7A**), suggesting that they could have a different receptor specificity. Competition experiments show that Δ*dtpA* single mutants are significantly resistant to CT^O91:H8^, though full resistance still requires disruption of both *dtpA* and *dtpB* (**Fig 8D**).

### Ionophore cytoplasm entry domains are modular

**Fig 9:**
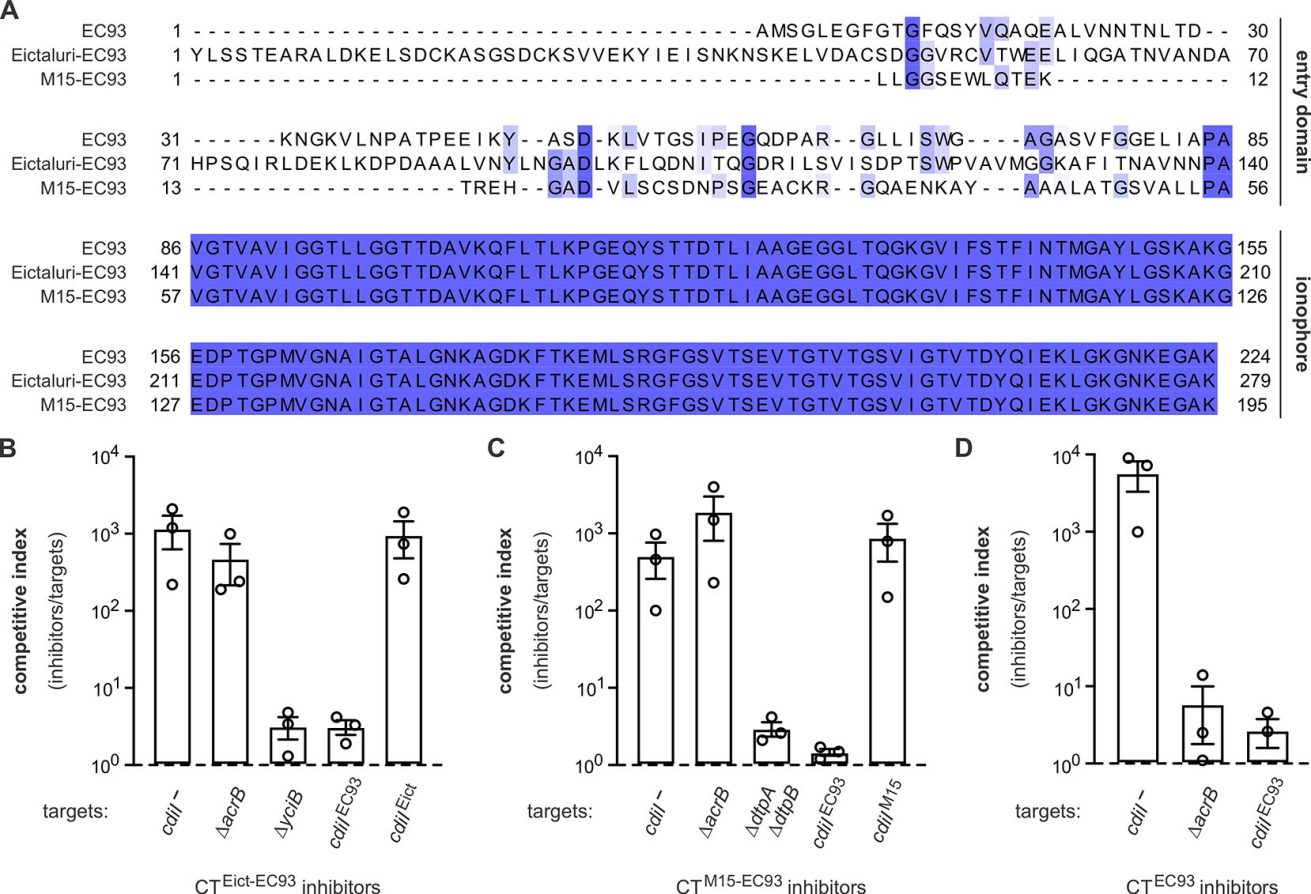
Hybrid ionophore toxins are functional. **A**) Alignment of CT^EC93^ with engineered hybrid toxins that contain the ionophore domain of CT^EC93^ fused to entry domains from CT^Eict^ and CT^M15^. Inhibitor strains that deploy CT^Eict-EC93^ (**B**), CT^M15-EC93^ (**C**), and CT^EC93^ (**D**) were co-cultured at a 1:1 ratio with the indicated target bacteria for 3 h. Viable inhibitor and target cells were enumerated and competitive indices calculated as the final ratio of inhibitor to target bacteria divided by the initial ratio. Presented data are the average ± standard error for three independent experiments.

AlphaFold2 modeling indicates that entry and ionophore domains are joined by flexible linkers, which suggests that the domains may function as modular units. To test this hypothesis, we generated hybrid CTs in which the AcrB-dependent entry domain of CT^EC93^ is replaced with entry domains from CT^Eict^ and CT^M15^ (**Fig 9A**). The resulting CT^EC93-Eict^ and CT^EC93-M15^ hybrid toxins inhibit the growth of target bacteria in competition co-cultures (**Fig 9B** and **9C**), though they are not as potent as wild-type CT^EC93^ (**Fig 9D**). Each hybrid toxin inhibits by virtue of the CT^EC93^-derived ionophore domain, because target cells are protected when they express *cdiI*^EC93^, but not *cdiI*^Eict^ or *cdiI*^M15^ (**Fig 9B** and **9C**). This latter result is consistent with AlphaFold2 predictions that immunity proteins interact primarily with the ionophore domain (**S2 Appendix**). We also tested the hybrid toxins against Δ*acrB*, Δ*yciB*, and Δ*dtpA* Δ*dtpB* target cells, and found that growth inhibition activity is dependent on the cognate membrane protein receptor (**Fig 9B, 9C** and **9D**). These findings indicate that the ionophore domain of CT^EC93^ can be assembled into target-cell membranes through alternative pathways dictated by the N-terminal entry domain.

### Membrane protein receptors are required for toxic activity

**Fig 10:**
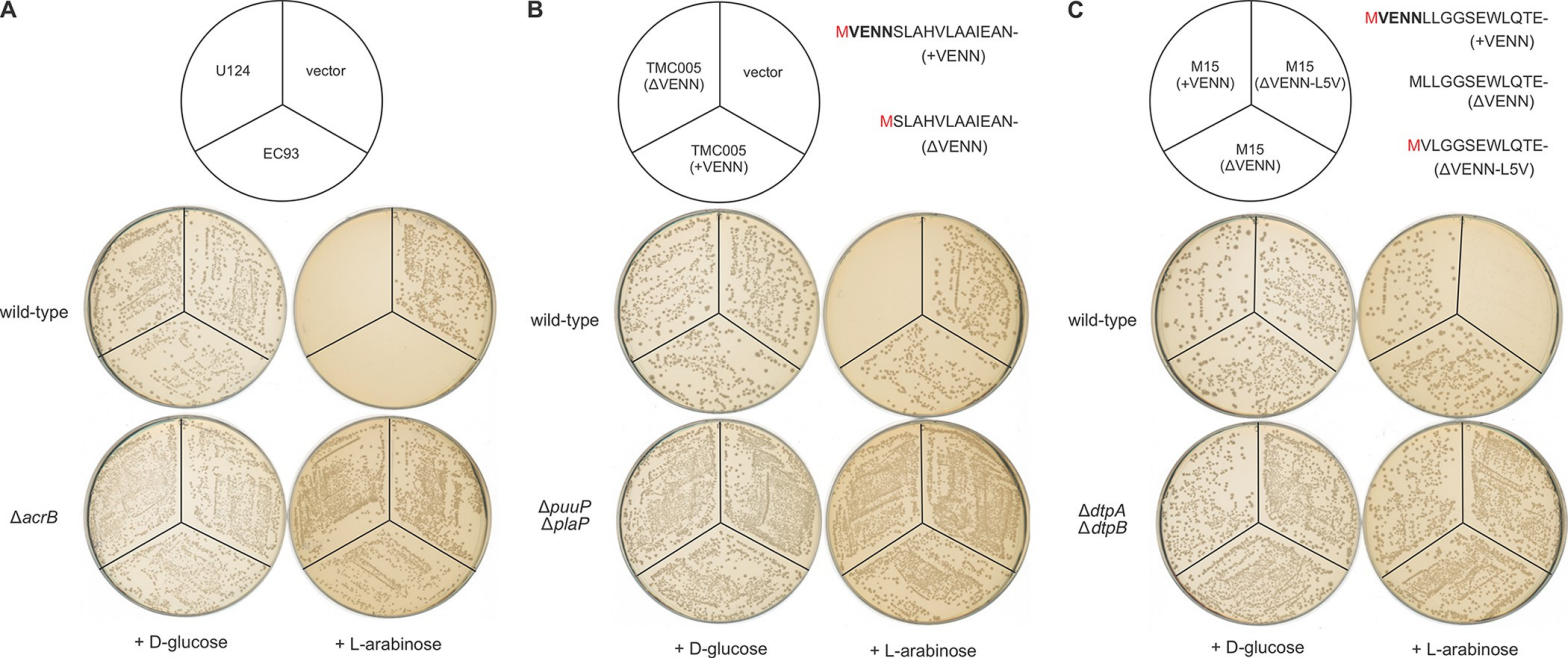
*E*. *coli* cells are inhibited by cytosolic expression of CDI ionophore toxins. **A**) Wild-type and Δ*acrB* cells were transformed with arabinose-inducible CT^EC93^ and CT^U124^ expression constructs, then plated on selective media supplemented with either D-glucose or L-arabinose. **B**) Wild-type and Δ*puuP* Δ*plaP* cells were transformed with arabinose-inducible CT^TMC005^ expression constructs, then plated on selective media supplemented with either D-glucose or L-arabinose. The initiating Met residues depicted in red are removed by methionine N-peptidase activity. **C**) Wild-type and Δ*dtpA* Δ*dtpB* cells were transformed with arabinose-inducible CT^M15^ expression constructs, then plated on selective media supplemented with either D-glucose or L-arabinose. The initiating Met residues depicted in red are removed by methionine N-peptidase activity, whereas the Met residue from the ΔVENN construct is not removed.

During CDI-mediated delivery, ionophore toxins are presumably assembled into the target-cell membrane from the periplasmic space (see **Fig 1B**). However, prior work has shown that CT^EC93^ is toxic when produced in the cytosol [11,39], suggesting that the ionophore can also insert from the cytoplasmic face of the cell membrane. To test whether AcrB is required for cytosolic toxicity, we compared the transformation efficiency of an arabinose-inducible CT^EC93^ expression plasmid into *acrB*^*+*^ and Δ*acrB* cells. The CT^EC93^ construct can be introduced into *E*. *coli acrB*^+^ cells in the absence of arabinose, but no transformants are obtained when the same cell mixture is plated onto arabinose-supplemented medium (**Fig 10A**). In contrast, the CT^EC93^ construct is maintained stably in *E*. *coli* Δ*acrB* mutants under both repressing and inducing conditions (**Fig 10A**). Similar results were obtained with an arabinose-inducible expression construct for the group 3 AcrB-dependent ionophore toxin from *E*. *coli* U124 (**Fig 10A**).

To determine whether other ionophores are toxic when produced in the cytosol, we generated expression plasmids for group 5 CT^TMC005^ and group 6 CT^M15^ toxins. Surprisingly, neither construct inhibits wild-type cell growth under inducing conditions (**Fig 10B** and **10C**). We previously found that a CT^EC93^ expression construct lacking the conserved N-terminal VENN sequence is more potent than one that includes the motif [11]. The latter observation suggests that additional N-terminal residues may interfere with ionophore assembly and/or activity. Accordingly, we found that a ΔVENN version of the CT^TMC005^ construct inhibits wild-type cell growth under inducing conditions (**Fig 10B**). ΔVENN-CT^TMC005^ expression has no effect on the growth of Δ*puuP* Δ*plaP* mutants (**Fig 10B**), again indicating that toxin activity is receptor-dependent. Deletion of the VENN motif from the CT^M15^ expression plasmid did not yield a toxic construct (**Fig 10C**). We reasoned that the ΔVENN-CT^M15^ construct may lack activity because the initiating Met residue cannot be removed post-translationally when the +2 residue is Leu (**Fig 10C**). Therefore, we replaced the Leu residue with Val to enable Met removal by methionine aminopeptidase. The resulting ΔVENN-L5V-CT^M15^ construct inhibits wild-type cell growth, with smaller colonies obtained on arabinose-supplemented medium (**Fig 10C**). Once again, toxicity is receptor-dependent, because ΔVENN-L5V-CT^M15^ expression has no effect of the growth of Δ*dtpA* Δ*dtpB* mutants (**Fig 10C**). Thus, membrane receptors are required for ionophore activity when normal delivery is bypassed with internal expression. Further, these results indicate that the N-terminus of the entry domain must be processed precisely for maximal toxicity.

## Discussion

Toxin diversity is a cardinal feature of CDI [1,11]. In a previous survey, we identified 20 toxin types carried by *E*. *coli* CdiA effectors [12]. The results of the current study reveal that five of those originally delineated toxin types share a similar structure and ionophoric activity with CT^EC93^. Based on these new findings, we now recognize 21 toxin types in the *E*. *coli* CdiA-CT repertoire, with the α-helical ionophores consolidated into one clade (type 10) (**Table 2**). Most CDI toxins from *E*. *coli* are nucleases, though the uncharacterized type 15 and type 17 toxins are predicted to have NAD^+^/NADH glycohydrolase and cytidine deaminase activities, respectively (**Table 2**). Many *E*. *coli* CdiA proteins also carry C-terminal Ntox25 domains (type 14), which were originally predicted to have RNase activity [13], but have recently been shown to disrupt the membrane potential [14]. Although Ntox25 domains have the same activity as the α-helical ionophores described here, AlphaFold2 predicts that the former toxin family adopts a distinct tapered β-barrel fold (**S10A Fig**). We initially failed to unify the type 10 α-helical ionophores because this family is exceptionally diverse compared to other CT toxin types. For example, CT domains from any given nuclease family typically share >90% sequence identity, whereas the type 10 ionophores exhibit only ~20 to 39% identity across the major groups (see **S1 Fig**). The type 14/Ntox25 ionophores are also quite variable, with some members sharing 29–39% pair-wise sequence identity. This radical diversification indicates that ionophore-immunity protein pairs are inherently robust to mutational changes. Nuclease toxin evolution may be more restrained because these enzymes must retain the ability to bind DNA/RNA substrates and catalyze phosphodiesterase/hydrolase reactions. We also note that the α-helical ionophore domains are fused to a greater variety of entry domains than other toxin types (**Table 2**). Most other *E*. *coli* CDI toxins enter target bacteria using one or two types of cytoplasm entry domain (**Table 2**). The results presented here show that type 10 α-helical ionophores collectively exploit seven different receptor proteins, further underscoring the functional plasticity of these toxins.

**Table 2:** *E*. *coli* CdiA-CT toxin types and cytoplasm entry domains.

toxin type	toxin domain annotation	toxin activity	entry domain type/receptor*	*E*. *coli isolate*	CdiA effector (GenBank)	references
1	DUF769	unknown	#11—unknown	DEC9E	EHW54111.1	
2	none	RNase	#11—unknown	97.0246	EIG93024.1	
3	none	unknown	MetI	EC1738	EIP59427.1	
4	CdiA-CT_Ec-like (cd20686) EndoU (PF14436)	tRNA anticodon nuclease (Glu)	PtsG	STEC_O31	EJK94116.1	[46]
5	none	tRNA acceptor stem nuclease (Glu/Asp)	PtsG	NC101	WFA97165.1	[36,44]
6	Endonuclease NS_2 (PF13930)	DNase	MetI	1303	AJF58502.1	
			DtpA/DtpB	HVH 98	ESK02023.1	
7	COG5529	RNase	SbmA	O32:H37 P4	EIF16908.1	
8	DUF4258 (PF14076)	tRNase (?)	#16—unknown	FHI89	WP_044686884.1	
			#11—unknown	117	WP_077898481.1	
9	CdiA-CT_Ec-like (cd20723)	tRNA acceptor stem nuclease (Ile)	PtsG	3006	EKI34460.1	[36,47]
10	none	ionophore	AcrB	EC93	AAZ57198.1	[3,6]
			AcrB	STEC2074	KYV32123.1	
			SecY	B799	EIG47241.1	[49]
			DtpA/DtpB	M15	APL26259.1	
			PuuP/PlaP	TMC005	WP_253030753.1	
			YciB	B088	EFE64162.1	
11	CdiA-CT_Ec-like (cd20700) Ntox28 pfam15605	tRNA anticodon nuclease (non-specific)	FtsH	536	ABG72516.1	[36,43]
			#26—unknown	PNUSAE019052	EEV7341131.1	
12	none	tRNA acceptor stem nuclease (Asn/Gln)	#13—unknown	EC869	EDU89581.1	
13	CDI_toxin_EC869_like (cd13444)	DNase	YciB	TA271	EGI36612.1	[36,41]
14	Ntox25 super family (cI21334) pfam15530	ionophore	AcrB	EC93	QNS35908.1	[14]
			AcrB	STEC_O31	EJK97217.1	[9]
			novel	Mt1B1	AVZ57724.1	
			novel	EC4	WP_039005204.1	
15	TNT (PF14021)	NAD glycohydrolase	PtsG	FCH1	WP_029488456.1	
16	PD-ExK motif	DNase (?)	SbmA	3-267-03_S3_C2	KDU01818.1	
17	MafB19 deaminase (PF14437)	cytosine deaminase	PtsG	STEC 2573	WP_077879060.1	
			SecY	696_ECOL	WP_049080366.1	
18	LHH/Endo VII	DNase	#16—unknown	PSU-0771	WP_078163250.1	
19	colicin D	tRNA anticodon nuclease (Arg)	#15—unknown	JE146-PJ18	HBC2943972.1	
20	cytotoxic (cI07564) pfam09000	16S rRNase	#15—unknown	NCTC9094	STE18217.1	
21	Peptidase_C39_like (cd02259)	cysteine peptidase	#15—unknown	TMP019540	HAV9700848.1	

*Entry domains with unknown receptors are numbered according to Bartelli *et al*. [40].

Membrane depolarization is a common growth-inhibition strategy, and several classes of pore-forming toxins are deployed during interbacterial conflict. The very first bacteriocin/microcin toxin described by Gratia in 1925 kills *E*. *coli* through membrane depolarization [58,59]. Microcin V (née colicin V) is an 88-residue peptide exported by some *E*. *coli* strains through a type 1 secretion mechanism. Although much smaller than CdiA, microcin V also relies on receptors in the outer and inner membranes to intoxicate target cells. Microcin V first binds to the iron-siderophore transporter Cir on the cell surface [60], which triggers Ton-dependent import to the periplasm. Subsequent integration into the cytoplasmic membrane requires the SdaC L-serine import protein [61]. Receptor dependencies have also been reported for ionophoric microcins E492 and H47, which hijack the mannose transporter and the F_o_ component of ATP synthase, respectively [62,63]. Nisin and related lantibiotics constitute another distinct class of peptide bacteriocins that depolarize bacterial membranes [64]. Lantibiotics inhibit Gram-positive bacteria but are generally inactive against Gram-negative species because the outer membrane prevents these peptides from gaining access to the cytoplasmic membrane. In addition, lantibiotics do not use protein receptors to enter the cell membrane and instead interact with lipid II [64], which is the precursor for peptidoglycan cell wall synthesis. The near universal conservation of lipid II across bacteria accounts for the broad-spectrum antimicrobial activity of lantibiotics. Conversely, bacteriocins that rely on protein receptors typically have narrow target-cell ranges, because bacterial cell-surface antigens are subject to positive selection and vary considerably between different species and strains [65].

Bacteria also release multi-domain bacteriocins to inhibit competitor cell growth. The most extensively studied of these are colicins, which use receptor-binding and translocation domains to deliver variable C-terminal toxin domains into *E*. *coli* cells [66]. The pore-forming domains of colicins A, B, E1, Ia, K and N have different sequences, yet all adopt the same 10-helix structure with eight amphipathic helices surrounding a central hydrophobic pair [66]. This globular structure imparts solubility and enables the colicin to diffuse through the environment until it encounters a susceptible bacterium. Receptor recognition then initiates either Tol- or Ton-dependent uptake of the colicin across the outer membrane [66]. Once inside the periplasm, interactions with the anionic surface of the cell membrane induce the pore-forming domain to unfold partially into a molten globule [67]. In this state, the hydrophobic helices evert from the domain core and initiate membrane insertion. Full integration requires the transmembrane potential [68], the polarity of which restricts pore assembly to the electropositive face of the membrane. As a consequence, pore-forming colicins are not toxic when expressed in the cytosol [69–71], but are able to form active channels when directed to the periplasm with signal peptides [71,72]. Thus, colicin insertion is receptor-independent and relies instead on the physicochemical properties of the cytoplasmic membrane. Pore-forming colicins also differ from the CDI toxins in their ionophoric activity. Depolarizing colicins do not disrupt the proton chemical gradient and instead form voltage-gated cation channels that mediate K^+^ efflux from the cell [73–75].

Type 6 secretion systems (T6SSs) mediate another form of contact-dependent competition between Gram-negative bacteria. The T6SS apparatus is a multi-protein machine that functions like a contractile bacteriophage [1,76]. T6SS^+^ bacteria assemble a contractile sheath around a central tube structure that contains effector proteins. The tube is capped with a phage-like tail-spike protein that also carries toxic effectors. Upon contraction of the sheath, the tube is ejected through the T6SS trans-envelope complex, and nearby cells are perforated by the spike-tipped projectile. This mechanism allows toxins to be transferred directly into the periplasm of Gram-negative target bacteria. The H1-T6SS of *Pseudomonas aeruginosa* deploys two membrane-depolarizing effectors, Tse4 and Tse5 [77,78]. Tse4 was initially proposed to contain an α-helical glycine-zipper motif, but AlphaFold2 modeling suggests that it instead forms a β-barrel resembling the Ntox25 domain (https://alphafold.ebi.ac.uk/entry/Q9I069) (**S10B Fig**). Tse5 is a large rearrangement hotspot (Rhs) repeat protein that encapsulates a C-terminal pore-forming domain [79,80]. The cryo-electron microscopy structure of Tse5 was recently reported, though the toxin domain is not resolved in the model [81]. Additionally, Coulthurst and coworkers discovered Ssp6 as a novel pore-forming T6SS effector from *Serratia marcescens* Db10 [82]. Like the depolarizing colicins, Tse4, Tse5 and Ssp6 all form cation-selective channels [77,80,82]. Tse4 and Ssp6 are also not toxic when over-produced in the *E*. *coli* cytosol and only form open channels when exported to the periplasmic space [77,79,82]. In contrast, the C-terminal pore-forming domain of Tse5 is toxic when expressed inside the cytosol [78,80], though the full-length effector shows a preference for assembly from the periplasmic side of the membrane [81].

The cytosolic toxicity of CDI ionophores is unusual because most other depolarizing toxins must be integrated into the membrane from the periplasmic space. Although microcin E492 is active when produced in the cytoplasm [62], other enterobacterial toxins like microcin V are only toxic when directed into the periplasm [61,83]. Cytosolic toxicity is perplexing because it depends on the same receptors that are hijacked during normal delivery. Given that CDI toxins are released into the target-cell periplasm (see **Fig 1B**, step 4) [5,39], entry domains are thought to bind periplasmic epitopes on their receptors. Therefore, it is difficult to envision how entry domains could also specifically recognize the cytoplasmic surface of these same receptors. This toxicity could be due to a small fraction of ionophore escaping into the periplasm through transient breaches in the membrane, though this has not been observed with overexpressed colicins or T6SS effectors. It is also possible that CDI ionophores carry cryptic secretion signals, but such signals appear superfluous given that the toxins are deposited directly into the target-cell periplasm [5,39]. Alternatively, cytosolic toxicity could reflect physiologically relevant delivery. Garcia and coworkers recently showed that cytosolic loops on the GltJK ABC transporter are required for entry of a *Burkholderia* CDI nuclease toxin [37]. Their findings suggest that entry domains may first penetrate the membrane in a receptor-independent manner, then induce translocation through interactions with the cytoplasmic surface of the receptor.

Although receptor-dependent ionophore assembly is not fully understood, it is clear that proteolytic processing of the entry domain is critical for toxin activity. Inclusion of the conserved VENN sequence abrogates CT^M15^ and CT^TMC005^ toxicity, though these additional residues have less of an effect on AcrB-dependent CT^EC93^ and CT^U124^ toxins [11]. CT^M15^ is particularly dependent on processing, because retention of even one additional N-terminal Met residue blocks its activity. Presumably, unprocessed toxins do not recognize their receptors or are otherwise unable to assemble into the membrane. These results suggest that the ionophores are inert in the context of full-length CdiA and only become activated upon proteolytic release. Given that CDI toxins are stowed within the inhibitor-cell periplasm before deployment (**Fig 1B**) [5,40,50], regulated CT processing could serve a quality control function that prevents futile auto-delivery. CdiA-CT cleavage is normally induced after transfer into the target-cell periplasm, and this proteolysis is required for subsequent translocation to the cytosol (**Fig 1B**, steps 4 & 5) [5]. CdiA-CT release is most likely mediated by the adjacent pretoxin domain (**Figs 1A** and **1B**) [5], though it is unclear how auto-processing is regulated to occur only after transfer into target bacteria. We note that precise cleavage after the VENN motif is also critical for CDI nuclease activity, because improperly processed tRNase toxins are unable to enter the target-cell cytoplasm [40]. Collectively, these observations suggest that auto-processing induces a conformational switch that enables entry domains to interact with their receptors and/or penetrate membrane bilayers.

## Materials & Methods

### Sequence analyses and structure predictions

Residues Ala2909 –Lys3132 of CdiA^EC93^ (NCBI: AAZ57198.1) were used to query the NCBI nonredundant protein sequence database with PSI-BLAST. The initial search recovered group 1, 2 and 3 toxins at high confidence, and a subset of group 4 and 6 ionophores were identified with low confidence (**S1 Appendix**). Secondary searches using group 4 CT^B088^ (NCBI: EFE64162.1) and group 6 CT^RHB08-C4-1^ (NCBI: MBA8481725.1) sequences led to the identification of group 5 toxins (**S1 Appendix**). The CT^RHB08-C4-1^ search also recovered several Endonuclease NS_2 toxins with DtpAB-dependent entry domains (**S1 Appendix**). Similarly, the CT^B088^ search identified DNase toxins with YciB-dependent entry domains (**S1 Appendix**). Multiple sequence alignments were used to group CdiA proteins with identical CTs, enabling the identification of 89 unique toxin sequences. CdiI sequences were predicted from open-reading frames found immediately downstream of the *cdiA* genes. In some instances, *cdiI* immunity genes are not annotated (or misannotated), though all ionophoric *cdiA* genes are associated with short open-reading frames that encode hydrophobic peptides. CT and CdiI sequences were aligned with Clustal Omega, and alignments rendered using Jalview (version: 2.11.3.3) [84] with conserved residues shaded at 30% sequence identity threshold. The resulting alignment was visualized as an unrooted circular tree using the interactive Tree of Life site (https://itol.embl.de/). The structures of representative CT•CdiI complexes were modeled with AlphaFold2 multimer (ColabFold v1.5.5: AlphaFold2 using MMseqs2) using default settings [35]. All structural models were rendered using PyMOL (version 2.5.4). The highest ranked AlphaFold2 models (based on local Distance Difference Test scores) (**S2 Fig**) were submitted to the European PDB ePISA website (https://www.ebi.ac.uk/pdbe/pisa/) to identify potential hydrogen-bond, salt-bridge and van der Waals interactions between the CT and CdiI proteins (**S2 Appendix**).

### Bacterial strains and growth

Bacterial strains used in this study are outlined in **S2 Table**. Bacteria were cultured at 37°C in lysogeny broth (LB) or on LB agar. Where appropriate, media were supplemented with antibiotics at the following concentrations: ampicillin (Amp) 150 μg mL^-1^, kanamycin (Kan) 50 μg mL^-1^, spectinomycin (Spm) 100 μg mL^-1^, chloramphenicol (Cm) 33 μg mL^-1^, rifampicin (Rif) 200 μg mL^-1^, tetracycline (Tet) 15 μg mL^-1^. The Δ*puuP*, Δ*plaP*, Δ*dtpA*, Δ*dtpB*, Δ*dtpC*, Δ*yciB*, Δ*acrB* and Δ*wzb* deletion alleles were obtained from the Keio collection [85] and transferred into *E*. *coli* MG1655 Δ*wzb* Δ*araBAD*::*spec* [86] cells by bacteriophage P1 mediated transduction. For construction of strains with multiple deletion mutations, kanamycin resistance cassettes were removed by FLP recombinase expression from plasmid pCP20 [87]. The *galK* linked dTomato expression system was described previously [49].

### Plasmid constructions

All plasmids are listed in **S3 Table**, and oligonucleotide primers are listed in **S4 Table**. The *E*. *coli* EC93 *cdiBAI* gene cluster was expressed from its native promoter elements on plasmid pCH1285, in which codons Ser2892-Ala2893 of *cdiA*^EC93^ are mutated to introduce a unique NheI site. Plasmid pDAL660(Δ1–39) [2] was amplified with oligonucleotides CH4262 and CH4842, and the fragment used as a megaprimer in a second PCR with oligonucleotide DL1527. The resulting product was digested with SphI/XbaI and ligated to SphI/AvrII-digested pDAL660(Δ1–39). The *cdiBAI*^EC93^ operon was then subcloned using BamHI and XhoI restriction sites into pET21(ΔHpa-Nhe) to generate plasmid pCH1285. The *cdiA-CT/cdiI*_o10_ module from *E*. *coli* EC869 was amplified from plasmid pCH10166 [49] with CH4262/CH2363 and the product ligated to pCH1285 via NheI/XhoI sites to generate plasmid pCH8274. All other *cdiA-CT/cdiI* gene pairs were synthesized as NheI/XhoI fragments by Twist Bioscience (South San Francisco, USA) for direct ligation to pCH1285. The *cdiA-CT/cdiI* modules from *E*. *coli* TMC005 and M15 were also amplified with primers CH3053/CH3954 and CH4127/CH4128 (respectively) for fusion to *cdiA*^EC93^ using a recombineering approach as previously described [41]. Upstream and downstream homology regions were amplified from *cdiA*^EC93^ with DL1527/DL2470 and DL1663/DL2368 (respectively), and these fragments were joined to the *cdiA-CT/cdiI* modules by overlap extension-PCR with primers DL1527/DL2368. The resulting products were electroporated together with plasmid pCH10163 into *E*. *coli* DY378 cells [41]. Recombinant plasmids were selected on yeast extract glucose agar supplemented with chloramphenicol and 10 mM D/L-*p*-chlorophenylalanine.

The *cdiI*^EC93^ and *cdiI*_o10_^EC869^ immunity genes were amplified with CH4929/CH29 and CH2362/CH2363 (respectively), digested with KpnI/XhoI and ligated to pCH405Δ to generate plasmids pCH1287 and pCH8268. All other immunity genes were amplified with specific forward primers in conjunction with a common CH6253 reverse primer (**S4 Table**), and products ligated to pCH405Δ via KpnI/XhoI restriction sites. The *cdiI*^TMC005^ and *cdiI*^M15^ immunity genes were also ligated to plasmid pTrc99aKX. Genes encoding membrane receptor proteins were PCR amplified and ligated to derivatives of plasmid pTrc99a for complementation studies: *dtpA* was amplified with CH5141/CH5477 and ligated via EcoRI/XbaI; *dtpB* was amplified with CH5366/CH4885 and ligated via KpnI/XhoI; *dtpC* was amplified with CH5367/CH4887 and ligated via KpnI/XhoI; *puuP* was amplified with CH4529/CH5085 and ligated via NcoI/XbaI; *plaP* was amplified with CH5095/CH5096 and ligated via KpnI/XhoI; and *yoeI-plaP* was amplified with CH6274/CH5096 and ligated via KpnI/XhoI. CdiA-CT coding sequences were amplified and placed under the control of the L-arabinose inducible promoter on plasmid pCH450 [88] for internal expression studies. CT^U124^ was amplified with CH4284/CH4302; CT^TMC005^ was amplified with CH4110/CH4111 (+VENN) and CH4237/CH4111 (ΔVENN); and CT^M15^ was amplified with CH4108/CH4109 (+VENN), CH4235/CH4109 (ΔVENN) and CH6269/CH4109 (ΔVENN-L5V).

Hybrid ionophore *cdiA-CT/cdiI* constructs were generated using overlap-extension PCR. A fragment encoding the EC93 ionophore domain and immunity protein was amplified using primers CH6270/CH29. Entry domain coding sequences were amplified from pCH8275 (*E*. *ictaluri*) with CH4262/CH6271 and pCH8278 (M15) with CH4262/CH6273. Fragments were used as templates for a second amplification with primers CH4263/CH29. The final products were digested with NheI/XhoI and ligated to pCH1285 to generate plasmids pCH8279 and pCH8281.

### Competition co-cultures

Inhibitor and target cell strains were grown to mid-log phase then adjusted to an optical density at 600 nm (OD_600_) of 3.0 in LB media. Strains were mixed at a 1:1 ratio and plated onto LB agar for co-culture at 37°C. Cells were harvested with a sterile swab into 1x M9 salts, and serial dilutions were plated onto selective media to enumerate viable cells for both populations. Competitive indices were calculated as the ratio of inhibitors to targets at 3 h divided by the initial ratio. Presented data are the averages ± standard error for three independent experiments.

### Mutagenesis and CDI^R^ selections

Transposon mutant libraries were constructed as described previously [36]. The *mariner* transposon was introduced into MG1655 by conjugation with MFD*pir*^+^ donor cells carrying pSC189 [89,90]. Donors were grown in LB medium supplemented with 30 μM diaminopimelic acid, then plated with MG1655 recipients on LB agar at 37°C for 4–5 h. Cell mixtures were harvested on a 0.22 μM nitrocellulose membrane into 0.5 mL of 1x M9 minimal medium, then plated onto Kan-supplemented LB agar to select transposon mutants. Each mutant pool was harvested into 1 mL of 1x M9 minimal media for subsequent selection by competition co-culture with inhibitor strains. Inhibitors strains carrying pCH6770 or pCH6771 were co-cultured with each of the six *mariner* mutant pools in 25 mL of LB media at 37°C. Co-cultures were seeded at a 1:2 ratio of inhibitor to target bacteria to minimize bottle-necking during the initial selection. After 3 h, co-cultures were plated onto LB agar supplemented with Kan to enumerate viable target cells and isolate survivors for further selection by iterative competition co-culture. After three rounds of co-culture, individual colonies were isolated and their resistance phenotypes assessed. Transposon insertion sites were determined by rescue cloning. Approximately 1 μg of genomic DNA from each transposon mutant was digested with NspI overnight. The restriction enzyme was heat-inactivated at 65°C for 25 min, and the reactions supplemented with 1 mM ATP and T4 DNA ligase for overnight incubation at 16°C. The ligation reactions were transformed into *E*. *coli* DH5ɑ *pir*^*+*^ cells. Transposon insertion sites were identified by DNA sequence analysis with primer CH2260.

Six independent pools of *E*. *coli* MG1655 Δ*wzb* cells carrying plasmid pZS21::*bamA* were irradiated with UV light at 260 nm at 15 mJ/cm^2^. Irradiated cells were diluted into 90 mL of Kan-supplemented LB media for recovery overnight in the dark. Each UV mutant pool was split in two and co-cultured at a 2:1 ratio with inhibitor cells that deploy CT^TMC005^ (pCH6771) and CT^M15^ (pCH6770). Inhibitors and target cells were adjusted to OD_600_ ~ 33, and 100 μL of the mixed cell suspension was plated on pre-warmed 8.5 cm LB-agar plates. After 3 h at 37°C, cells were harvested into 1 mL of 1x PBS and serially diluted to enumerate viable target cell counts. Surviving kanamycin-resistant target bacteria were used in a second round of selection in co-cultures with inhibitor cells. After the third round of selection, individual target cell clones were isolated for whole-genome sequencing.

### Whole-genome sequencing

Genomic DNA was prepared from CT^TMC005^ and CT^M15^ resistant isolates using Genomic-tip 100/G columns (Qiagen). PCR-free library construction and whole-genome re-sequencing was performed by the BGI sequencing facility (Hong Kong, China) using the HiSeq 4000 PE150 sequencing system (Illumina Inc., San Diego, CA, USA). Illumina analysis pipeline was used for image analysis, base calling and quality score calibration. Raw sequence reads were filtered and exported as FASTQ files. Sequence reads were mapped onto the *E*. *coli* MG1655 genome using the CLC Genomics workbench (Qiagen), and SNP/DIP/structural variant detection analyses were used to identify unique mutations in the isolates.

### Flow cytometry

Dissipation of the membrane potential was monitored with bis(1,3-dibutylbarbituric acid) trimethine oxonol [DiBAC_3_(4)] staining [49,51]. CDI inhibitor strains were co-cultured with dTomato-labeled target bacteria at a 1:1 ratio with a starting OD_600_ of 1.0. After 1 h, 150 μL aliquots were removed and treated with 10 μg mL^-1^ DiBAC_3_(4). After 30 min incubation in the dark, cells were collected by centrifugation and washed with 1x PBS for flow cytometry (MACSQuant, Miltenyi Biotech). To normalize the data from the co-cultures, a stop gate was applied to each sample based on the fluorescence intensity of a monoculture of Tomato^+^ target bacteria. 100,000 events were recorded in the gated population for each sample and analyzed for DiBAC_3_(4) uptake by fluorescence intensity in the FITC channel.

### Growth inhibition by internal ionophore expression

Competent *E*. *coli* strains were prepared using TSS solution (10% polyethylene glycol-8000, 30 mM MgCl_2_, 5% dimethylsulfoxide in LB medium) as described [91]. Purified plasmid DNA (100 ng) was transformed into competent cells, followed by recovery in 1.0 mL of LB media for 1 h at 37°C. 25 μL of each transformation cell suspension was plated onto LB agar supplemented with tetracycline and either D-glucose or L-arabinose.

## Supporting information

S1 Fig Alignment of CDI ionophore toxins from *E*. *coli* isolates. CdiA-CT sequences from the proteins listed in S1 Table were aligned using Clustal Omega. The alignment was rendered using Jalview (version: 2.11.3.3) with conserved residues shaded at 30% sequence identity threshold. Groups are indicated along the right and the putative glycine zipper motif is indicated with asterisks (*).
(TIF)

S2 Fig AlphaFold2 modeling of CT•CdiI complex structures. The structures of representative cognate CT•CdiI pairs were modeled using AlphaFold2 multimer. Output sequence coverage, local distance difference test (lDDT) and predicted aligned error (pae) plots for the computational models are presented. For all pae plots, chain A corresponds to the CT toxin, and chain B is the CdiI immunity protein.
(TIF)

S3 Fig Group 2 ionophore toxin-immunity protein sequences. Group 2 CdiA-CT (panel **A**) and CdiI (panel **B**) sequences from the proteins listed in S1 Table were aligned using Clustal Omega. Alignments were rendered using Jalview (version: 2.11.3.3) with conserved residues shaded at 30% sequence identity threshold. Secondary structure elements are indicated above each alignment, with entry domain helices depicted in violet and ionophore helices in red. Residues predicted to interact with cognate partners are highlighted with red boxes.
(TIF)

S4 Fig Group 3 ionophore toxin-immunity protein sequences. Group 3 CdiA-CT (panel **A**) and CdiI (panel **B**) sequences from the proteins listed in S1 Table were aligned using Clustal Omega. Alignments were rendered using Jalview (version: 2.11.3.3) with conserved residues shaded at 30% sequence identity threshold. Secondary structure elements are indicated above each alignment, with entry domain helices depicted in violet and ionophore helices in red. Residues predicted to interact with cognate partners are highlighted with red boxes.
(TIF)

S5 Fig Groups 4 ionophore toxin-immunity protein sequences. CdiA-CT (panel **A**) and CdiI (panel **B**) sequences from the proteins listed in S1 Table were aligned using Clustal Omega. Alignments were rendered using Jalview (version: 2.11.3.3) with conserved residues shaded at 30% sequence identity threshold. Secondary structure elements are indicated above each alignment, with entry domain helices depicted in violet and ionophore helices in red. Residues predicted to interact with cognate partners are highlighted with red boxes.
(TIF)

S6 Fig Group 5 ionophore toxin-immunity protein sequences. **A**) Group 5 CdiA-CT sequences from S1 Table were aligned with selected group 6 CdiA-CTs that share homologous ionophore domains. **B**) Group 5 CdiI sequences from S1 Table were aligned with selected group 6 immunity proteins using Clustal Omega. Alignments were rendered using Jalview (version: 2.11.3.3) with conserved residues shaded at 30% sequence identity threshold. Secondary structure elements are indicated above each alignment, with entry domain helices depicted in violet and ionophore helices in red. Residues predicted to interact with cognate partners are highlighted with red boxes.
(TIF)

S7 Fig Group 6 ionophore toxin-immunity protein sequences. Group 6 CdiA-CT (panel **A**) and CdiI (panel **B**) sequences from the proteins listed in S1 Table were aligned using Clustal Omega. Alignments were rendered using Jalview (version: 2.11.3.3) with conserved residues shaded at 30% sequence identity threshold. Secondary structure elements are indicated above each alignment, with entry domain helices depicted in violet and ionophore helices in red. Residues predicted to interact with cognate partners are highlighted with red boxes.
(TIF)

S8 Fig PuuP and PlaP protein sequences. PuuP (NCBI: WP_000996856.1) and PlaP (NCBI: WP_000019197.1) from *E*. *coli* MG1655 were aligned using Clustal Omega. Alignments were rendered using Jalview (version: 2.11.3.3) with conserved residues shaded at 30% sequence identity threshold.
(TIF)

S9 Fig Dtp protein sequences. DtpA (NCBI: WP_000100932.1), DtpB (NCBI: WP_001098652.1), DtpC (NCBI: WP_000856829.1) and DtpD (NCBI: WP_001032689.1) from *E*. *coli* MG1655 were aligned using Clustal Omega. Alignments were rendered using Jalview (version: 2.11.3.3) with conserved residues shaded at 30% sequence identity threshold.
(TIF)

S10 Fig AlphaFold2 models of Ntox25 and Tse4 ionophore toxins in complex with cognate immunity proteins. **A**) The Ntox25-containing CdiA-CT from *E*. *coli* EC93 (NCBI: WP_061892950.1/QNS35908.1) and its cognate CdiI immunity protein (NCBI: QNS35909.1) were modeled using AlphaFold2 multimer. The Ntox25 domain is rendered in red, the putative AcrB-dependent entry domain in violet and the immunity protein in green. **B**) Tse4 (NCBI: WP_003099160.1) and Tsi4 (NCBI: WP_003114429.1) from *P*. *aeruginosa* PAO1 were modeled using AlphaFold2 multimer. Tse4 is rendered in red and Tsi4 in green.
(TIF)

S1 Table CDI ionophore and immunity protein accession numbers. (XLSX)

S2 Table Bacterial strains used in this study. (XLSX)

S3 Table Plasmids used in this study. (XLSX)

S4 Table Oligonucleotides used in this study. (XLSX)

S1 Appendix PSI-BLAST search outputs. Recovered CdiA proteins are listed with toxin group annotations. Proteins highlighted in Figs 2A and **S1** are indicated in boldface.
(XLSX)

S2 Appendix ePISA outputs. Intermolecular contacts were predicted using the ePISA server and outputs annotated as individual spreadsheets. Polar contacts are indicated in orange and van der Waals interactions in cyan. Predicted secondary structure elements are indicated and color coded as in the main and supplemental figures.
(XLSX)
