# Correction: Contact-dependent growth inhibition (CDI) systems deploy a large family of polymorphic ionophoric toxins for inter-bacterial competition

**DOI:** 10.1371/journal.pgen.1011922

**Published:** 2025-10-24

**Authors:** The PLOS Genetics Staff

Fig 7E is incorrect. The publisher apologizes for the error. The authors have provided a corrected version here.

**Fig 7:**
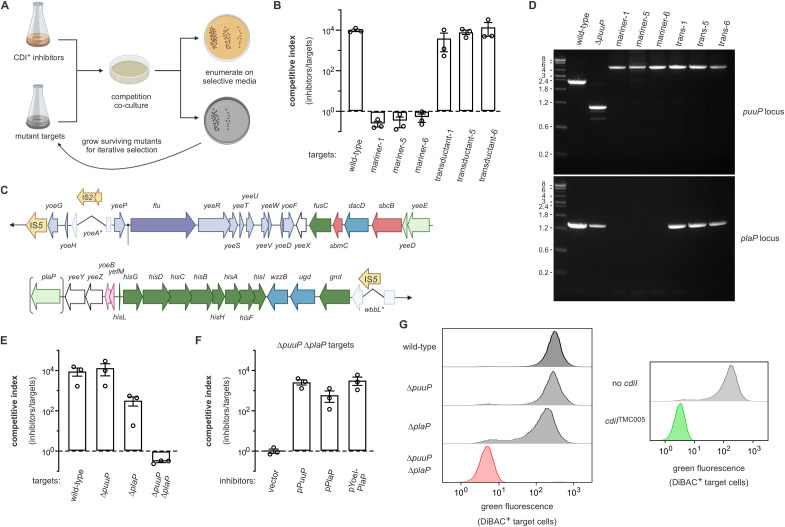
Group 5 ionophore toxins utilize paralogous putrescine import proteins as receptors. **A)** Selection scheme to isolate CDI-resistant mariner transposon insertion mutants. **B)** Transposon insertions in puuP were identified in selections for CTTMC005 resistant mutants, but puuP disruptions are not sufficient to confer resistance. Inhibitor strains that deploy CTTMC005 were co-cultured at a 1:1 ratio with the indicated target bacteria for 3 h. Viable inhibitor and target cells were enumerated and competitive indices calculated as the final ratio of inhibitor to target bacteria divided by the initial ratio. Presented data are the average ± standard error for three independent experiments. **C)** Genomic region deleted in CTTMC005 resistant mutants. **D)** PCR analyses of the puuP and plaP loci from CTTMC005 resistant mariner mutants. **E)** CTTMC005 exploits the paralogous putrescine transporters PuuP and PlaP as receptors. Inhibitor strains that deploy CTTMC005 were co-cultured at a 1:1 ratio with the indicated target bacteria for 3 h. Viable inhibitor and target cells were enumerated and competitive indices calculated as the final ratio of inhibitor to target bacteria divided by the initial ratio. Presented data are the average ± standard error for three independent experiments. **F)** Complementation of ΔpuuP ΔplaP mutants restores sensitivity to CTTMC005 intoxication. Inhibitor strains that deploy CTTMC005 were co-cultured at a 1:1 ratio with ΔpuuP ΔplaP target bacteria that carry the indicated plasmids for 3 h. Viable inhibitor and target cells were enumerated and competitive indices calculated as the final ratio of inhibitor to target bacteria divided by the initial ratio. Presented data are the average ± standard error for three independent experiments. **G)** ΔpuuP ΔplaP mutants are not depolarized by CTTMC005. The indicated red fluorescent target strains were incubated with inhibitors that deploy CTTMC005 for 1 h, then the cell suspensions were treated with DiBAC4 (3) to label depolarized cells for quantification by flow cytometry. The protection afforded by the cdiITMC005 immunity gene is also presented for comparison. Panels A and C were created in BioRender. Halvorsen, T. (2020) https://BioRender.com/o68v649.
