# Supplementary figures and images for: Contact-dependent growth inhibition (CDI) systems deploy a large family of polymorphic ionophoric toxins for inter-bacterial competition
Source: PLoS Genet. 2024 Nov 26;20(11):e1011494. doi: 10.1371/journal.pgen.1011494 (PMC11630599; doi:10.1371/journal.pgen.1011494)

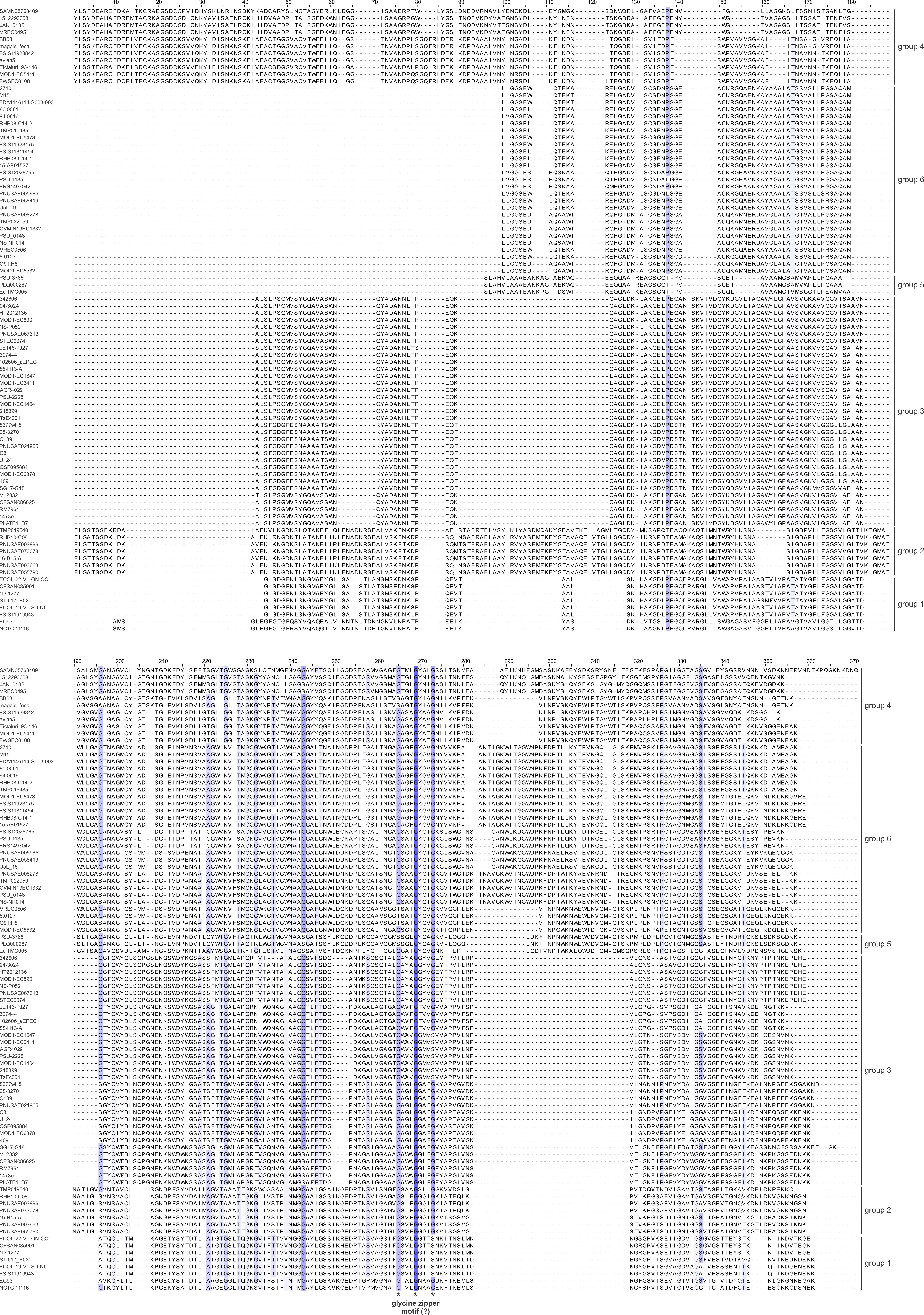

Supplement: S1 Fig — CdiA-CT sequences from the proteins listed in S1 Table were aligned using Clustal Omega. The alignment was rendered using Jalview (version: 2.11.3.3) with conserved residues shaded at 30% sequence identity threshold. Groups are indicated along the right and the putative glycine zipper motif is indicated with asterisks (*). (TIF) [file pgen.1011494.s001.tif]

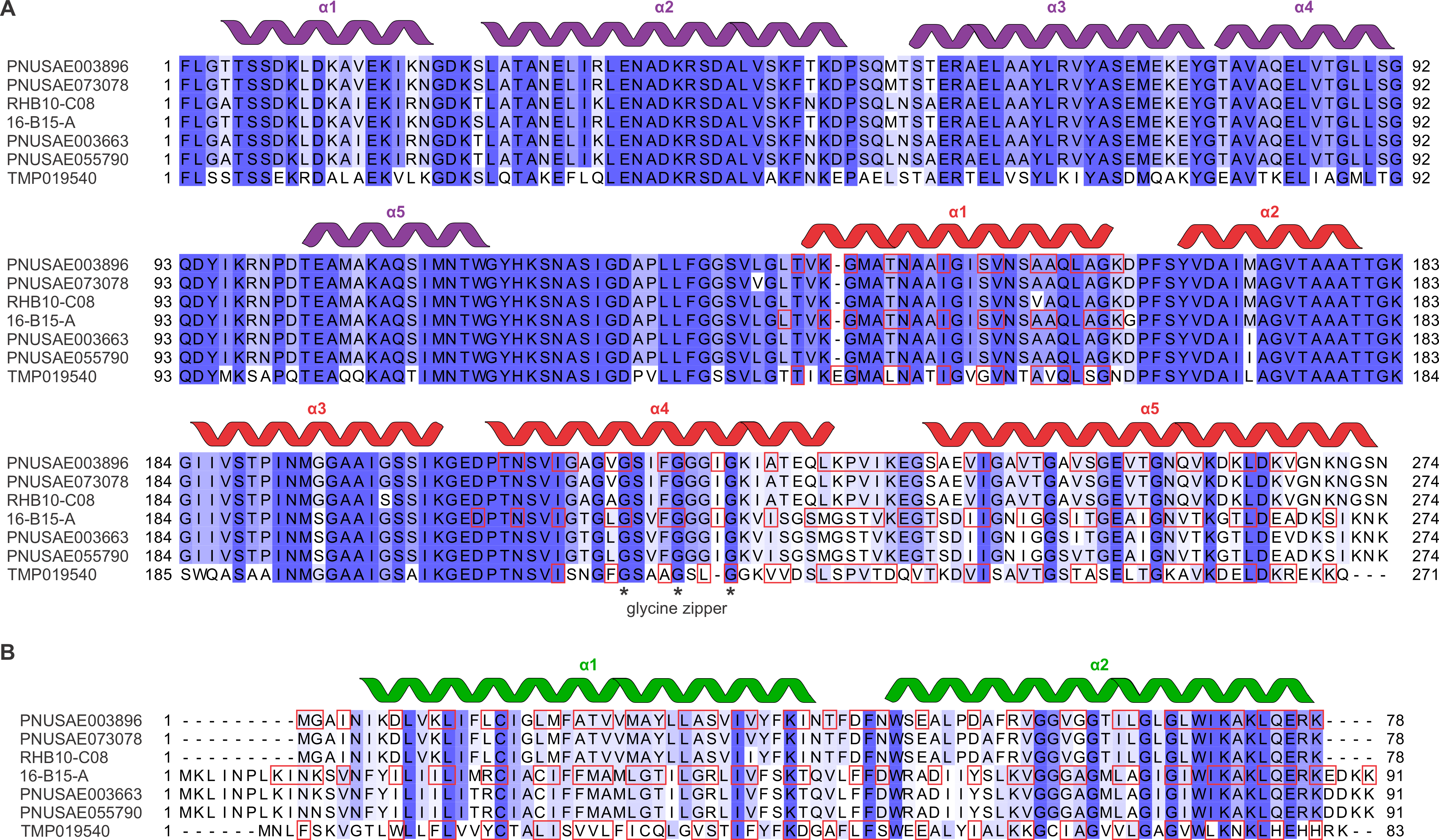

Supplement: S3 Fig — Group 2 CdiA-CT (panel A) and CdiI (panel B) sequences from the proteins listed in S1 Table were aligned using Clustal Omega. Alignments were rendered using Jalview (version: 2.11.3.3) with conserved residues shaded at 30% sequence identity threshold. Secondary structure elements are indicated above each alignment, with entry domain helices depicted in violet and ionophore helices in red. Residues predicted to interact with cognate partners are highlighted with red boxes. (TIF) [file pgen.1011494.s003.tif]

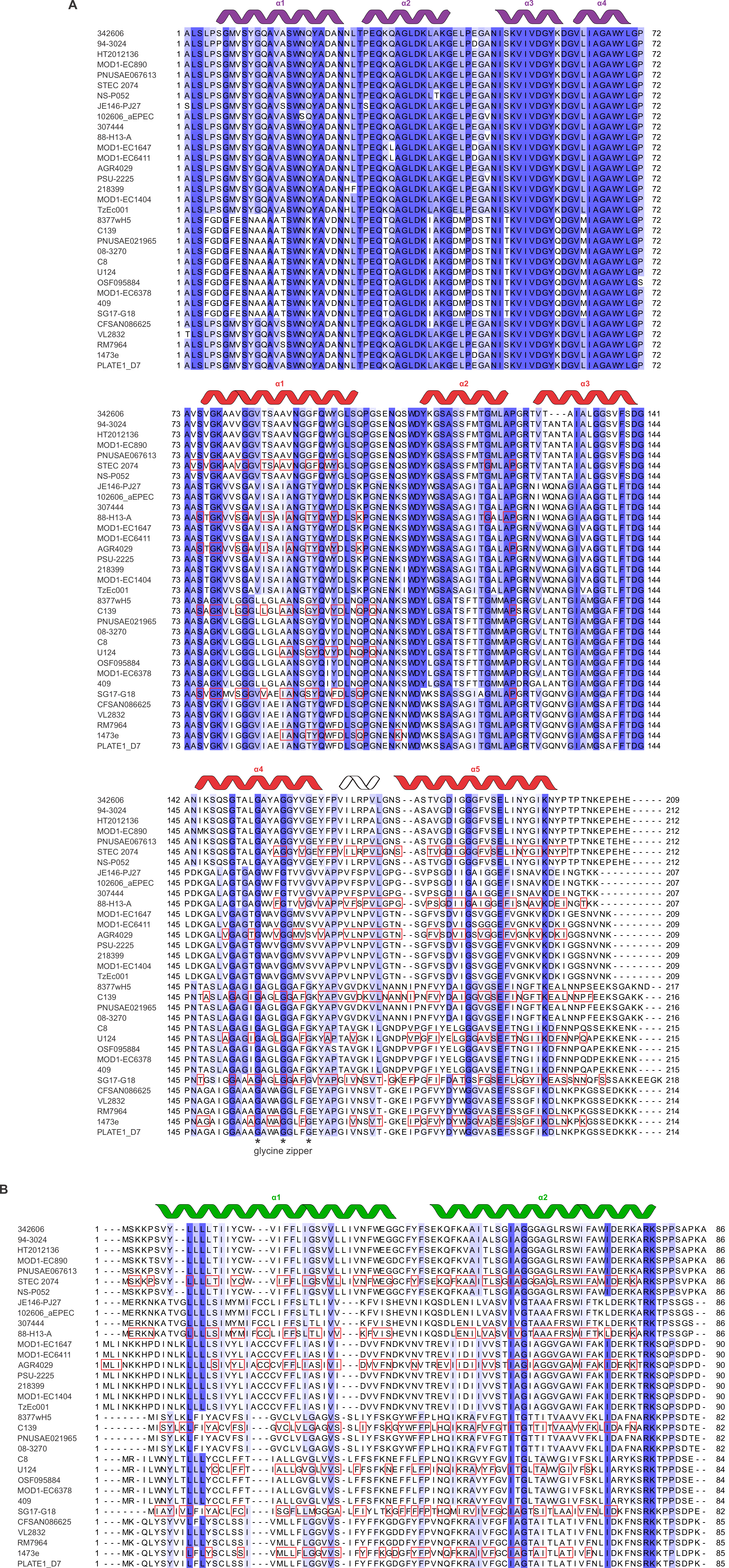

Supplement: S4 Fig — Group 3 CdiA-CT (panel A) and CdiI (panel B) sequences from the proteins listed in S1 Table were aligned using Clustal Omega. Alignments were rendered using Jalview (version: 2.11.3.3) with conserved residues shaded at 30% sequence identity threshold. Secondary structure elements are indicated above each alignment, with entry domain helices depicted in violet and ionophore helices in red. Residues predicted to interact with cognate partners are highlighted with red boxes. (TIF) [file pgen.1011494.s004.tif]

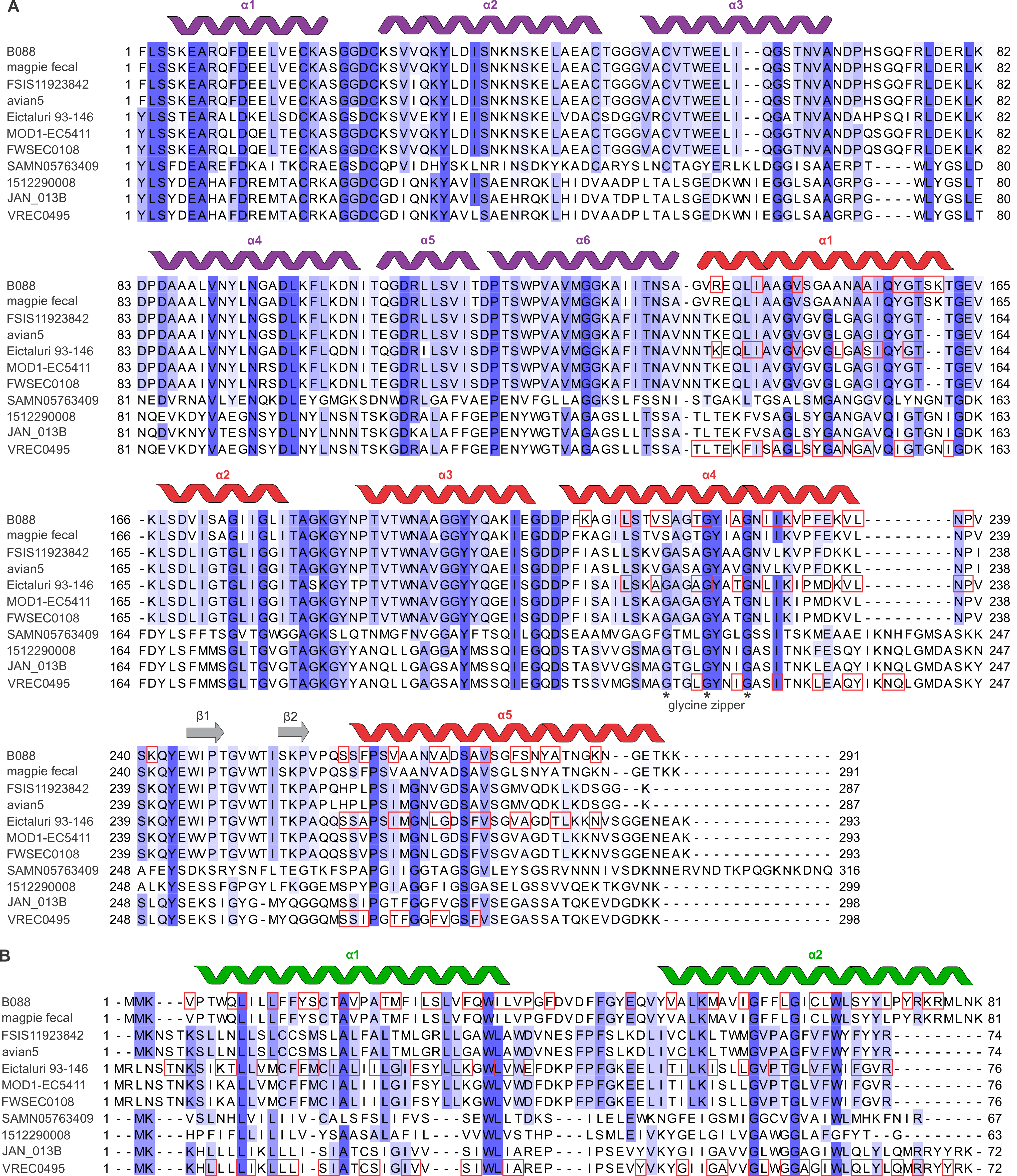

Supplement: S5 Fig — CdiA-CT (panel A) and CdiI (panel B) sequences from the proteins listed in S1 Table were aligned using Clustal Omega. Alignments were rendered using Jalview (version: 2.11.3.3) with conserved residues shaded at 30% sequence identity threshold. Secondary structure elements are indicated above each alignment, with entry domain helices depicted in violet and ionophore helices in red. Residues predicted to interact with cognate partners are highlighted with red boxes. (TIF) [file pgen.1011494.s005.tif]

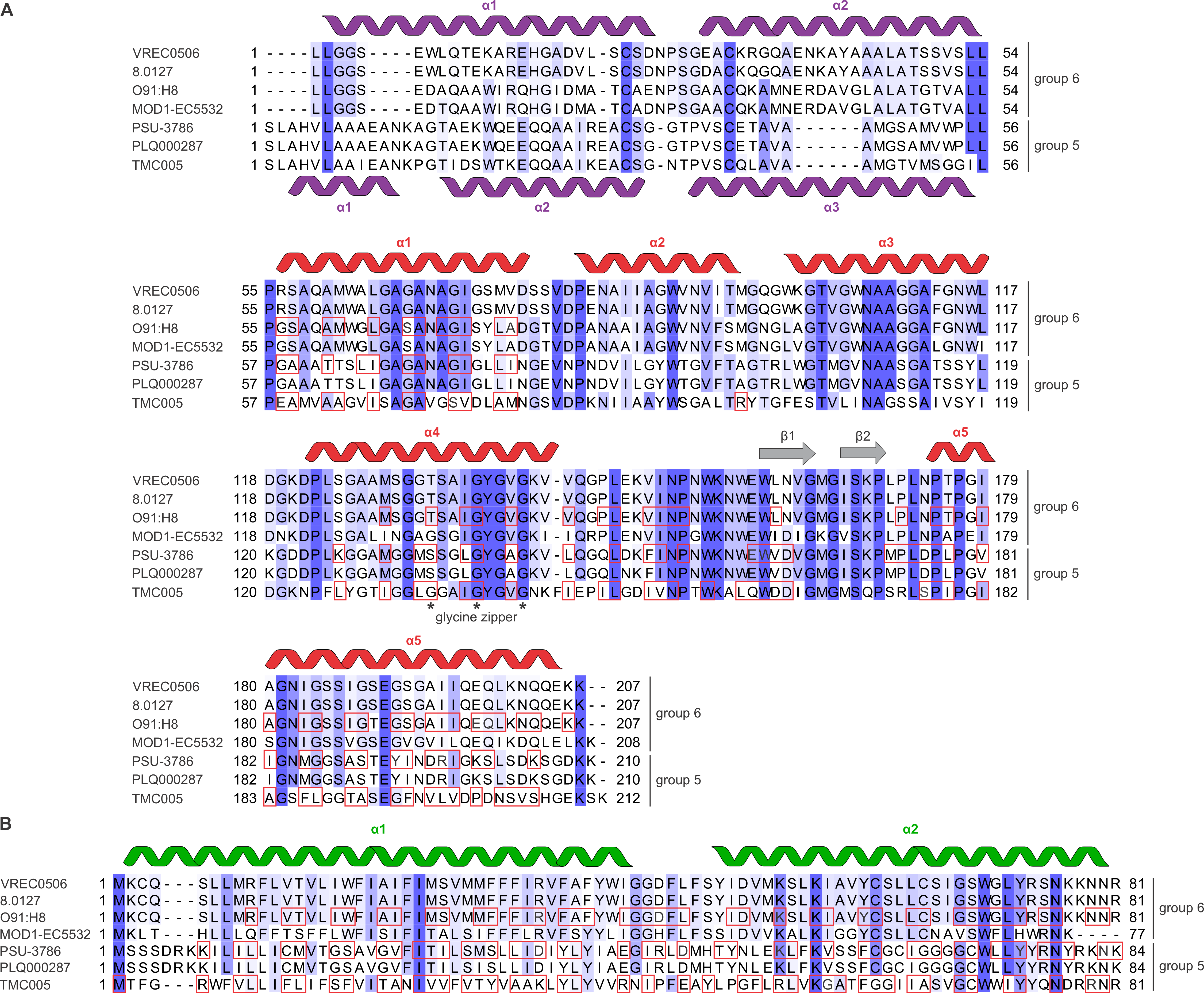

Supplement: S6 Fig — A) Group 5 CdiA-CT sequences from S1 Table were aligned with selected group 6 CdiA-CTs that share homologous ionophore domains. B) Group 5 CdiI sequences from S1 Table were aligned with selected group 6 immunity proteins using Clustal Omega. Alignments were rendered using Jalview (version: 2.11.3.3) with conserved residues shaded at 30% sequence identity threshold. Secondary structure elements are indicated above each alignment, with entry domain helices depicted in violet and ionophore helices in red. Residues predicted to interact with cognate partners are highlighted with red boxes. (TIF) [file pgen.1011494.s006.tif]

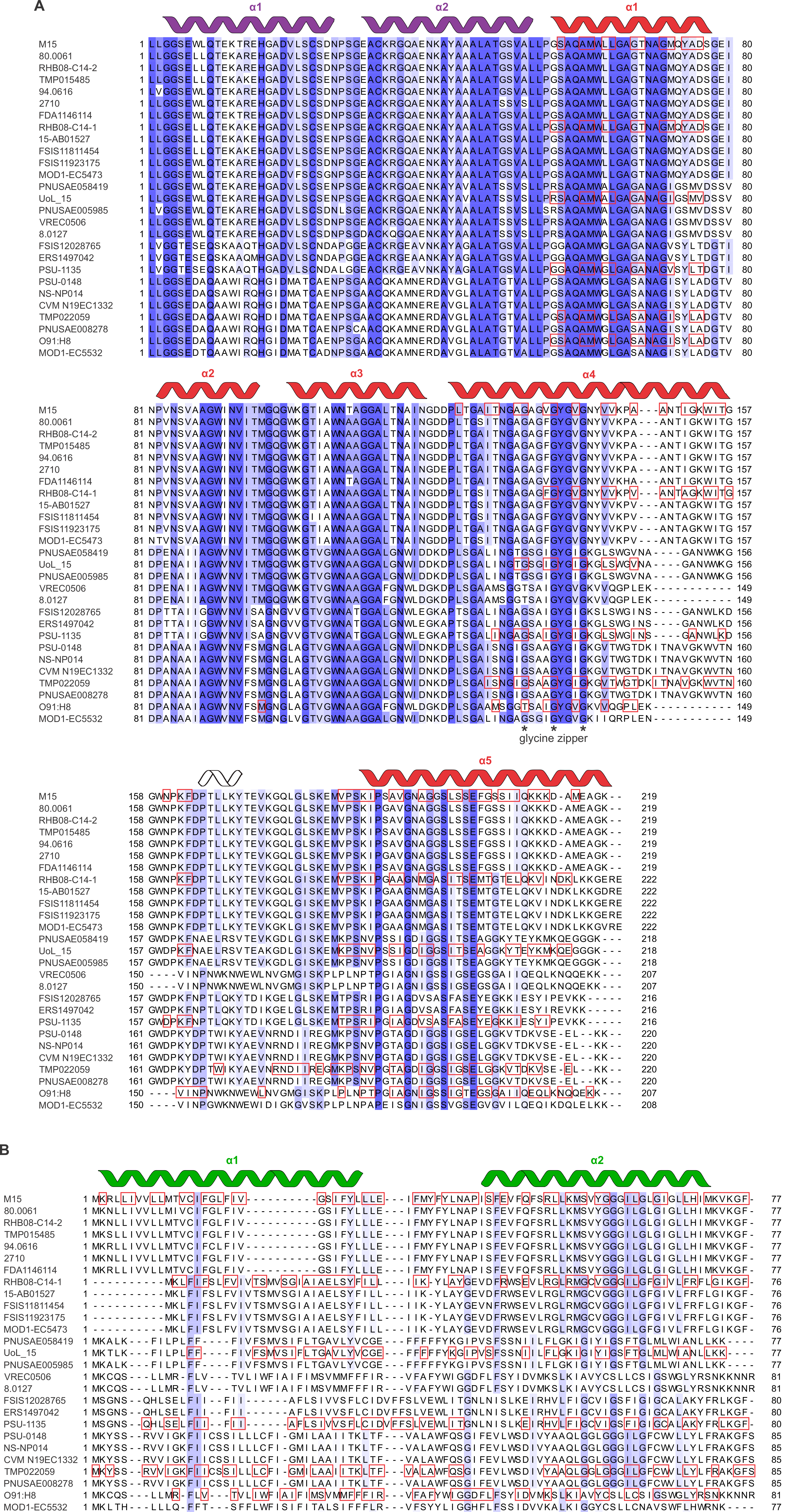

Supplement: S7 Fig — Group 6 CdiA-CT (panel A) and CdiI (panel B) sequences from the proteins listed in S1 Table were aligned using Clustal Omega. Alignments were rendered using Jalview (version: 2.11.3.3) with conserved residues shaded at 30% sequence identity threshold. Secondary structure elements are indicated above each alignment, with entry domain helices depicted in violet and ionophore helices in red. Residues predicted to interact with cognate partners are highlighted with red boxes. (TIF) [file pgen.1011494.s007.tif]

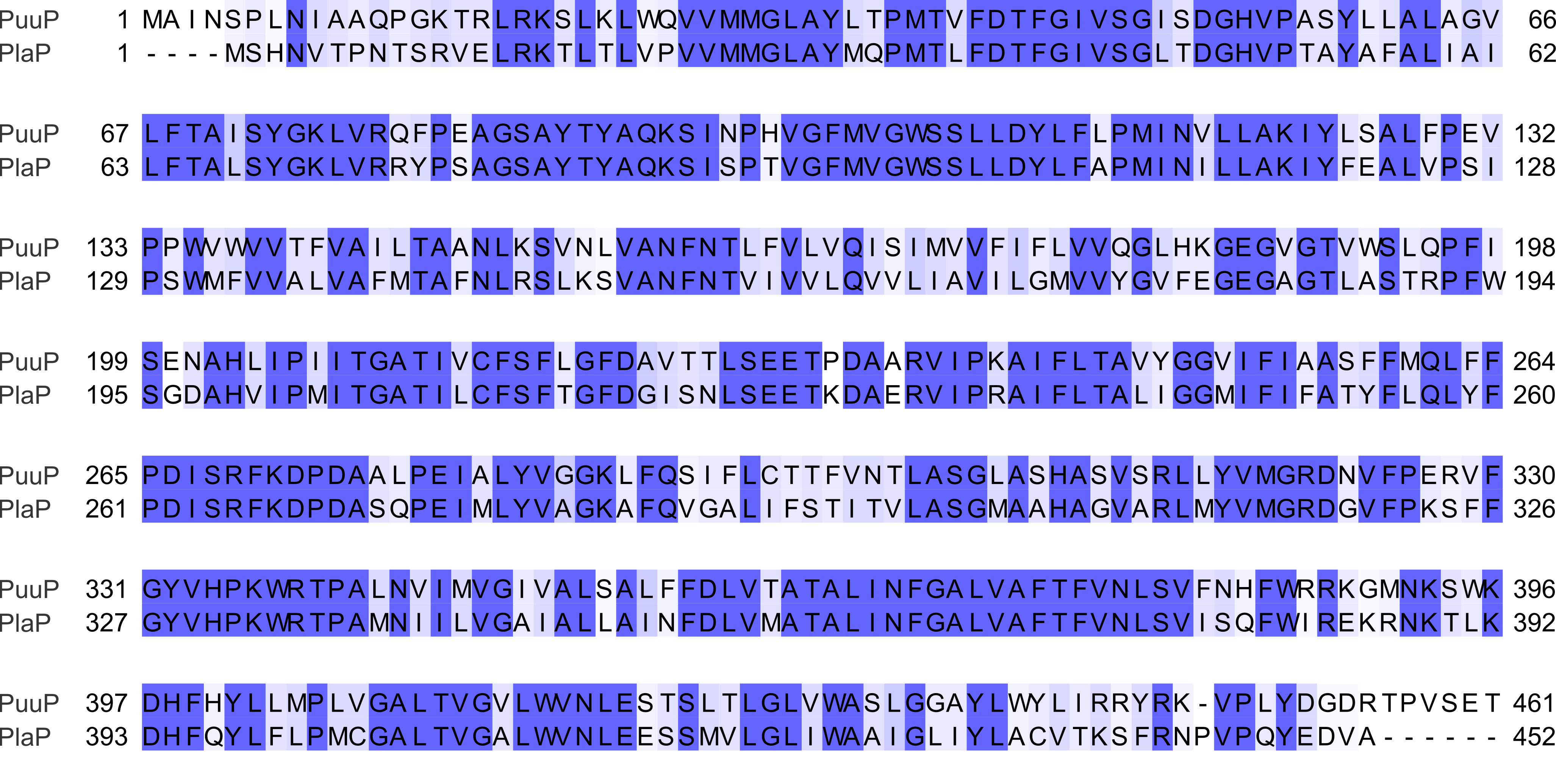

Supplement: S8 Fig — PuuP (NCBI: WP_000996856.1) and PlaP (NCBI: WP_000019197.1) from E. coli MG1655 were aligned using Clustal Omega. Alignments were rendered using Jalview (version: 2.11.3.3) with conserved residues shaded at 30% sequence identity threshold. (TIF) [file pgen.1011494.s008.tif]

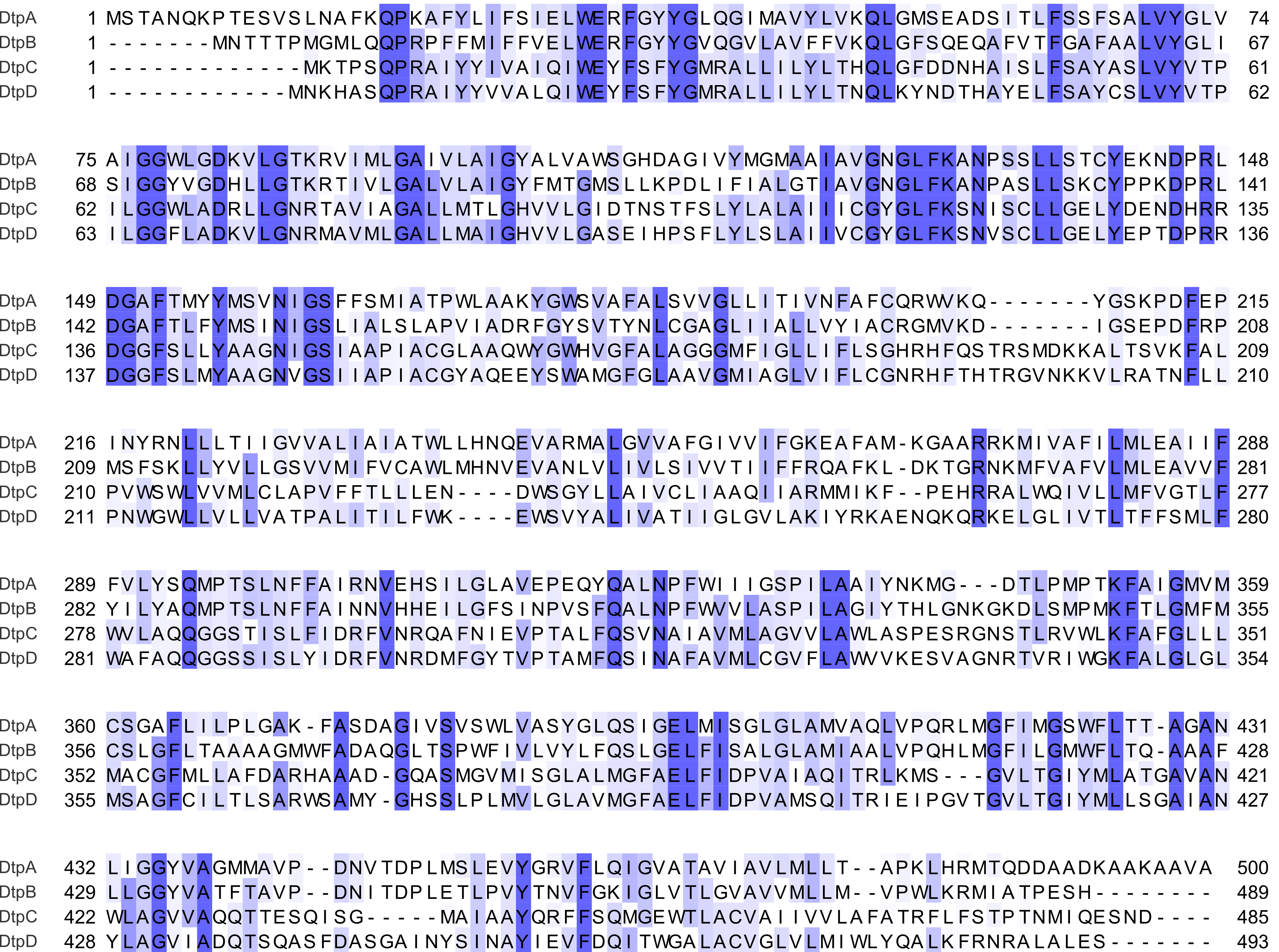

Supplement: S9 Fig — DtpA (NCBI: WP_000100932.1), DtpB (NCBI: WP_001098652.1), DtpC (NCBI: WP_000856829.1) and DtpD (NCBI: WP_001032689.1) from E. coli MG1655 were aligned using Clustal Omega. Alignments were rendered using Jalview (version: 2.11.3.3) with conserved residues shaded at 30% sequence identity threshold. (TIF) [file pgen.1011494.s009.tif]

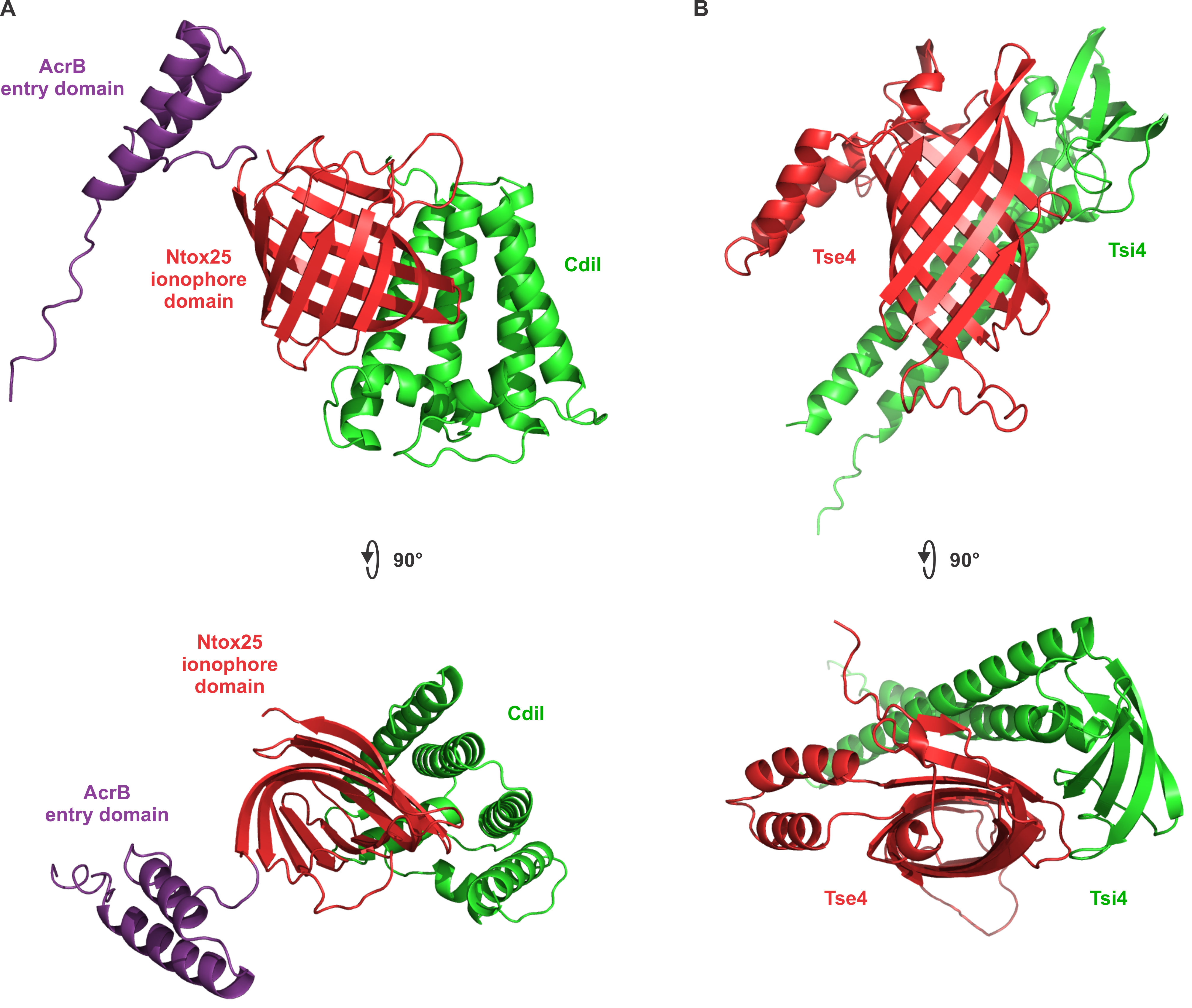

Supplement: S10 Fig — A) The Ntox25-containing CdiA-CT from E. coli EC93 (NCBI: WP_061892950.1/QNS35908.1) and its cognate CdiI immunity protein (NCBI: QNS35909.1) were modeled using AlphaFold2 multimer. The Ntox25 domain is rendered in red, the putative AcrB-dependent entry domain in violet and the immunity protein in green. B) Tse4 (NCBI: WP_003099160.1) and Tsi4 (NCBI: WP_003114429.1) from P. aeruginosa PAO1 were modeled using AlphaFold2 multimer. Tse4 is rendered in red and Tsi4 in green. (TIF) [file pgen.1011494.s010.tif]
